# Targeted Rapamycin Delivery via Magnetic Nanoparticles to Address Stenosis in a 3D Bioprinted in Vitro Model of Pulmonary Veins

**DOI:** 10.1002/advs.202400476

**Published:** 2024-05-02

**Authors:** Liqun Ning, Stefano Zanella, Martin L. Tomov, Mehdi Salar Amoli, Linqi Jin, Boeun Hwang, Maher Saadeh, Huang Chen, Sunder Neelakantan, Lakshmi Prasad Dasi, Reza Avazmohammadi, Morteza Mahmoudi, Holly D. Bauser‐Heaton, Vahid Serpooshan

**Affiliations:** ^1^ Wallace H. Coulter Department of Biomedical Engineering Emory University School of Medicine and Georgia Institute of Technology Atlanta GA 30322 USA; ^2^ Department of Mechanical Engineering Cleveland State University Cleveland OH 44115 USA; ^3^ Department of Biomedical Engineering Texas A&M University College Station TX 77843 USA; ^4^ J. Mike Walker ’66 Department of Mechanical Engineering Texas A&M University College Station TX 77840 USA; ^5^ Department of Radiology and Precision Health Program Michigan State University East Landing MI 48824 USA; ^6^ Department of Pediatrics Emory University School of Medicine Atlanta GA 30322 USA; ^7^ Children's Healthcare of Atlanta Atlanta GA 30322 USA; ^8^ Sibley Heart Center at Children's Healthcare of Atlanta Atlanta GA 30322 USA

**Keywords:** 3D bioprinting, endothelial cell proliferation, magnetic nanoparticles, perfusion bioreactor, Pulmonary vasculature, pulmonary vein stenosis, restenosis, targeted drug delivery

## Abstract

Vascular cell overgrowth and lumen size reduction in pulmonary vein stenosis (PVS) can result in elevated PV pressure, pulmonary hypertension, cardiac failure, and death. Administration of chemotherapies such as rapamycin have shown promise by inhibiting the vascular cell proliferation; yet clinical success is limited due to complications such as restenosis and off‐target effects. The lack of in vitro models to recapitulate the complex pathophysiology of PVS has hindered the identification of disease mechanisms and therapies. This study integrated 3D bioprinting, functional nanoparticles, and perfusion bioreactors to develop a novel in vitro model of PVS. Bioprinted bifurcated PV constructs are seeded with endothelial cells (ECs) and perfused, demonstrating the formation of a uniform and viable endothelium. Computational modeling identified the bifurcation point at high risk of EC overgrowth. Application of an external magnetic field enabled targeting of the rapamycin‐loaded superparamagnetic iron oxide nanoparticles at the bifurcation site, leading to a significant reduction in EC proliferation with no adverse side effects. These results establish a 3D bioprinted in vitro model to study PV homeostasis and diseases, offering the potential for increased throughput, tunability, and patient specificity, to test new or more effective therapies for PVS and other vascular diseases.

## Graphical abstract

Manufacturing a patient inspired A,B) 3D in vitro model of pulmonary veins using bioprinting C,D) together with drug‐loaded magnetic nanoparticles and perfusion bioreactor technologies E). The in vitro and in silico F) models serve as a robust platform to study endothelial cell response to microenvironment in the context of vascular stenosis, as well as targeted drug delivery and therapy G).

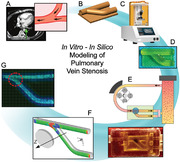

## 1 Introduction

Pulmonary vein stenosis (PVS) is a rare condition that is characterized by the progressive reduction in the lumen size of one or multiple pulmonary veins (PVs). Congenital PVS occurs in ≈0.4% of congenital heart diseases (CHDs).^[^ ^1^, ^2^ ^]^ In the pediatric patients, PVS is often an aggressive disease that is characterized by neointimal obstruction of the PVs, leading to pulmonary hypertension, right heart failure, and death.^[^ ^3^, ^4^ ^]^ In adults, acquired PVS is increasingly being reported following radiofrequency ablation for atrial fibrillation (1–1.5% incidence rate).^[^ ^5^, ^6^ ^]^ While post‐ablation PVS typically results in less morbidity and mortality than those reported in the congenital types, these acquired disease could still be highly symptomatic, associated with dyspnea and even hemoptysis.^[^ ^7^ ^]^

To date, treatment strategies for PVS include surgery, transcatheter intervention (e.g., balloon angioplasty and stent implantation), pharmacotherapies, and lung transplantation.^[^ ^8^, ^9^, ^10^ ^]^ These clinical efforts, however, face several major challenges, including the recurrence of PVS following anatomic intervention (i.e., restenosis), and the progression of the disease to previously unaffected vasculature.^[^ ^11^, ^12^ ^]^ Transcatheter stent implantation in stenotic PVs is often burdened with the development of in‐stent restenosis (37 ± 10% freedom from > 50% restenosis at 12 months^[^ ^13^ ^]^). Limited success and mixed results of the established treatment methods for PVS have motivated the research to develop new and/or more effective therapies. Studies on the pathogenesis of the intimal occlusive lesion in PVS have identified that intimal hyperplasia, mediated by the conversion/dedifferentiation of normal vascular cells (endothelial‐mesenchymal transition, EndMT^[^ ^14^ ^]^) and proliferation of myofibroblast‐like cells, is the primary mechanism of obstruction in the PVs.^[^ ^15^, ^16^, ^17^ ^]^ New therapies for PVS can be investigated through targeting and regulating the signaling pathways involved in such pathophysiological processes (e.g., tyrosine kinase blockade^[^ ^17^ ^]^).

Among various chemotherapy compounds, Sirolimus, a mammalian target of rapamycin (mTOR) inhibitor, has recently shown promise in treating PVS, either as prophylactic therapy or to reduce in‐stent stenosis, by targeting the vascular cell proliferation.^[^ ^18^, ^19^, ^20^ ^]^ Sirolimus (rapamycin) treatments in PVS patients, either by systemic administration or via drug‐eluting stents, are reported effective in slowing the neointimal proliferation in the PVs/stents and improving survival in infants and children with moderate to severe stenosis.^[^ ^20^, ^21^ ^]^ However, many of these patients with stents required reintervention, some developed atresia with others necessitating stent removal due to continued restenosis.^[^ ^20^ ^]^ Other concerns, such as immunosuppressive side effects, have been raised for higher doses of rapamycin therapies in PVS patients.^[^ ^20^ ^]^ Furthermore, prolonged rapamycin treatment has shown to reduce endothelial cell viability and function; however, the molecular and cellular mechanisms underlying the endothelial damage are not fully examined.^[^ ^22^, ^23^ ^]^

One of the key limitations that has hindered the in‐depth analysis of PVS mechanisms and therapies is the suboptimal experimental models, particularly in vitro platforms that could faithfully model the complex pathophysiology of the disease.^[^ ^24^, ^25^ ^]^ In this study, we utilized digital light processing (DLP)‐based 3D bioprinting technology, along with perfusion bioreactor systems to develop a novel robust in vitro platform to study PVS. Utilization of the bioprinting technology allows for fabrication of precise and tunable patient‐specific tissue models based on data obtained from medical imaging techniques such as MRI or CT scans in a replicable process, while the enhanced fabrication speed of DLP modality facilitates large‐scale manufacturing of 3D models enabling high‐throughput screening of different therapeutics. Further, conjugation of drug onto superparamagnetic iron oxide nanoparticles (SPIONs) was used to assess the targeted delivery of rapamycin to the site of injury (stenosis) via an external magnetic field. Bioprinted bifurcated PV‐like channels were endothelialized via human umbilical vein endothelial cells (HUVECs) and perfused under a physiological flow rate. Results from this work could establish a high‐throughput and highly tunable in vitro model, as a research‐enabling platform to tease out cellular and molecular mechanisms underlying PVS pathophysiology, as well as providing a unique opportunity to develop new or more effective pharmacotherapies for PVS and vascular defects.

## 2 Experimental Section

### 2.1 Materials

Gelatin powder derived from porcine skin (Type A, SLCC7838), methacrylic anhydride (MA), 2‐Hydroxy‐4′‐(2‐hydroxye‐thoxy)−2‐methylpropiophenone (Irgacure), poly(ethylene glycol) diacrylate (PEGDA), lithium phenyl‐2,4,6‐trimethylbenzoylphosphinate (LAP), and phosphate buffer saline (PBS) solution were all purchased from Sigma‐Aldrich (Wisconsin, US). AlamarBlue reagents were obtained from Bio‐Rad (Hercules, US). HUVEC culture medium (VascuLife VEGF) was purchased from Lifeline Cell Tech (Oceanside, US). Calcein‐acetoxymethylester (calcein‐AM) and propidium iodide (PI) were purchased from Biotium (Fremont, US). The antibodies for CD31, phosphohistone H3 (pH3), wheat germ agglutinin (WGA), and Ki67 were purchased from ThermoFisher (US). Rapamycin readymade solution in 2.74 mm DMSO (2.5 mg mL^−1^), materials for synthesis of the SPIONs (e.g., iron chlorides and sodium hydroxide) and poly (ethylene glycol)‐co‐fumarate (PEGF) were purchased from Sigma.

### 2.2 Preparation of GelMA Solution

Gelatin methacryloyl (GelMA) (Sigma) was produced following the established protocol.^[^ ^26^ ^]^ Briefly, porcine gelatin powder was dissolved in PBS (10% w/v) at 50 °C, followed by dropwise addition of MA (Sigma) and stirring at 50 °C for 3 h to functionalize the gelatin. Additional warm PBS was added to stop the reaction. Dialysis of the mixture was conducted using deionized water for 1 week at 40 °C with water exchange 3 times a day. Purified GelMA solution was lyophilized (7 days) and stored in dark at −20 °C until use. For bioprinting purposes, the lyophilized GelMA foam was reconstituted via sterile PBS to prepare a 10% w/v GelMA solution with 0.5% w/v Irgacure (Sigma). GelMA bioinks were stored in dark at 4 °C for 1–2 weeks prior use.

### 2.3 DLP Bioink Preparation

A novel optimized GelMA‐based bioink solution (CytoInk6000)^[^ ^27^, ^28^, ^29^ ^]^ was custom‐designed and prepared for DLP bioprinting to yield adequate fidelity.^[^ ^30^, ^31^, ^32^, ^33^ ^]^ Briefly, 10% (w/v) of porcine GelMA (Sigma) was mixed with cold water fish gelatin (10% w/v) (Sigma), PEGDA6000 (2.5% w/v) (Sigma), LAP photoinitiator (1% w/v), and 1X pen/strep to inhibit contamination. Mixed reagents were suspended in sterile PBS and incubated overnight at 37 °C while protected from light. The bioink was then adjusted to pH of ≈7.2–7.4. Next, 1.5 mm of Tartrazine was added to serve as a photoabsorber. The final bioink solution was incubated at 37 °C for another 3 h and then stored at 4 °C until use.

### 2.4 3D Model Design and DLP‐Based Bioprinting of 3D Pulmonary Vein (PV) Constructs

The design of 3D pulmonary vein (PV) channels was inspired by the bifurcated PV geometry from patient data and was created using SOLIDWORKS computer‐aided design (CAD) software (US). The created CAD model consisted of PV‐like channels at 4 and 2 mm outer and inner diameter, respectively and overall width and height of 14 and 25 mm, respectively. The CAD model was exported as an STL file, loaded into the Lumen X+ bioprinter (CELLINK, BICO, Sweden), and sliced for 3D printing at 100 µm layer height. Constructs were DLP printed according to user‐defined parameters, including a 40% exposure (≈10 mW cm^−2^), 8 sec per layer of exposure, 2X burn‐in, and a 100 µm first layer thickness. Following printing, PV constructs were transferred to a 12‐well tissue culture plate and submerged in (washed with) sterile PBS supplemented with antibiotics (2% v/v) to inhibit contamination. Constructs were stored in a tissue culture incubator for 3–5 days to allow for non‐cross‐linked bioink to leach out of the hydrogel samples. The PBS solution was changed 3–4 times per day. The use of deidentified patient imaging was considered non‐human subject research and could be utilized in this manner as determined by the Children's Healthcare of Atlanta Internal Review Board.

### 2.5 Bioprinting Fidelity Measurement

Geometrical features of the printed PV models, including the construct length (*l*), width (*w*), and height (*h*), as well as the channel diameter (*d*) and circularity (*c*) were measured (*n* = 3) as indices for printing fidelity as described before.^[^ ^34^ ^]^ These indices were defined as the ratio between the experimental (bioprinted) and the theoretical (CAD) values for each parameter, where the ratio of 1 indicates an ideal structural fidelity.

### 2.6 Mechanical Characterization of 3D Bioprinted PV Constructs

The elastic (Young's) modulus (*E*), which reflects the stiffness of bioprinted models, was measured using the microindentation test (Mach‐1, Biomomentum Inc., Quebec, Canada) and linear contact mechanics as described previously.^[^ ^26^, ^27^, ^28^, ^35^, ^36^ ^]^ Microindentation was conducted on the outer surface of bioprints (*n* = 6) and on the inner surface of the PV channels (*n* = 10). A 500 µm spherical indenter tip with an indenting depth of 100 µm at 2 µm s^−1^ was utilized. Stiffness (*S*) was calculated based on the slope of the linear trend line (initial 5–20%) in the force‐displacement unloading curves for each group. The reduced elastic modulus (*E_r_*) was then calculated using the following equation:^[^ ^37^ ^]^

(1) $$E_{r}=\frac{\sqrt{\pi }}{2\beta }\;\frac{S}{\sqrt{A\left(h_{c}\right)}}$$

where *A*(*h_c_*) is the projected contact area at the depth *h_c_*, and β  =  1 is a dimensionless constant obtained from the following equation:

(2) $$A\left(h_{c}\right)=2\pi Rh_{c}-\pi {h_{c}}^{2}$$

where

(3) $$h_{c}=h_{max}-\varepsilon \frac{P_{max}}{S}$$

where *P_max_* and *h_max_* are the maximum unloading force and displacement, respectively. The dimensionless ε constant = 0.75 for the spherical indenter used in this study.^[^ ^38^ ^]^ The Young's modulus, *E*, could be calculated using the following equation:^[^ ^37^ ^]^

(4) $$\frac{1}{E_{r}}=\frac{1-v^{2}}{E}+\frac{1-v_{i}^{2}}{E_{i}}$$

where the *E_i_* represents the elastic modulus of the indenter tip (= 2 GPa), and *v* and *v_i_* are the Poisson's ratio of the hydrogel (= 0.5) and the indenter tip material used (= 0.5), respectively.

### 2.7 Nanoparticle (NP) Synthesis and Characterization

SPIONs were synthesized using the co‐precipitation approach and coated with PEGF as described in detail in prior work.^[^ ^39^ ^]^ Briefly, deionized (DI) water containing NaOH (1.2 m) and PEGF were treated with bubbling argon gas for 30 min for deoxygenation; iron salts were dissolved in DI water containing 1 m HCl, and synthesis of SPIONs was started by dropwise addition of iron salt solutions to the NaOH solution under an argon atmosphere, and homogenization using high‐speed homogenizer. The resulting colloidal suspension (i.e., PEGF‐coated SPIONs) was then centrifuged at 10 000 g for 20 min and redispersed in DI water several times. The collected particles were then redispersed in rapamycin‐readymade solution and incubated for 1 h under homogenization. The final solution was introduced to magnetic activated cell sorting (MACS) system to collect the rapamycin‐entangled PEGF‐coated SPIONs while the rest of the solution was removed. Using high‐performance liquid chromatography (HPLC), the concentration of the rapamycin in the removed solution was measured (1.4 mg mL^−1^). The rapamycin‐entangled PEGF‐coated NPs (1.1 mg mL^−1^ rapamycin) were then redispersed in DI water. Finally, the unsaturated polyester coatings were cross‐linked by redox polymerization in the presence of ammonium persulphate ((NH_4_)_2_S_2_O_8_) as initiator system and (2‐dimethylamino) ethyl methacrylate (DMAEMA) as accelerator. The obtained ferrofluid was collected and washed three times with PBS. The collected rapamycin‐loaded cross‐linked PEGF‐SPIONs were reproduced in PBS and kept at 4 °C for future use.

The hydrodynamic size of the PEGF‐coated SPIONs and rapamycin‐loaded cross‐linked PEGF‐SPIONs and their size distribution were measured using dynamic light scattering (DLS) (Zetasizer nano series DLS instrument, Malvern Panalytical, UK).^[^ ^40^, ^41^ ^]^ A Helium‐Neon laser with a wavelength of 632 nm was used for the size distribution measurement at room temperature.

Transmission electron microscopy (TEM) analysis of the PEGF‐coated SPIONs and rapamycin‐loaded cross‐linked PEGF‐SPIONs were carried out using a JEM‐2200FS (JEOL Ltd., US) operated at 200Kv. The instrument was equipped with 7 an in‐column energy filter and an Oxford X‐ray energy dispersive spectroscopy (EDS) system. Twenty microliters of the PEGF‐coated SPIONs and rapamycin‐loaded cross‐linked PEGF‐SPIONs were drop‐casted onto the copper grid for TEM imaging.

### 2.8 HUVEC Culture and 2D NP‐Targeting Assays

HUVECs (Lifeline Cell Technology, US) were cultured in human EC medium (VascuLife VEGF medium complete kit, Lifeline) with 1% antibiotic (the Gibco Antibiotic‐Antimycotic solution, Gibco, US). Culture media was changed every 2 days. At 90% confluency, EC monolayers were trypsinized and passaged. Passage numbers ranging from 19 to 23 were used for all 2D and 3D cell culture assays. To passage the cells, HUVECs were washed with 10 mL of sterile PBS and 1 mL of 0.05% Trypsin/EDTA (Invitrogen, US) for 30 s. Trypsin was then aspirated and replaced with 5 mL of EC medium to fully detach cells with the help of pipetting.

For the 2D cell culture assays, after harvesting, HUVECs were transferred into 12‐well culture plates (4000 cells per well ≈1150 cells cm^−2^) and cultured for 7 days. Neodymium disc magnets were used, consisting of Ni‐Cu‐NiPlated, at 4.0 mm diameter and 2.4 mm thickness, which were magnetized axial (grade N40, D094A1, Amazing Magnets, US). Disc magnets were attached via clear tape to the external bottom surface of wells in a random position to accumulate (target) the SPIONs. On days 2 and 4, plain and rapamycin‐loaded SPIONs were added to 2D culture wells at a concentration of 100 mg mL^−1^. Culture medium without SPIONs was used for the control culture group. After 7 days of culture, cells were fixed via 10% formalin solution (VWR, US) for 15 min. After PBS washes (5 × 5 min), fixed cells were kept in fresh PBS and stored at 4 °C for further inspection.

### 2.9 Endothelialization of 3D Bioprinted PV Constructs

3D bioprinted vascular constructs were endothelialized as previously described.^[^ ^34^, ^36^, ^42^ ^]^ Briefly, HUVECs were manually injected into the PVs’ lumen (180 µL of cell suspension at 1 × 10^7^ cells mL^−1^), followed by incubation at 37 °C and 5% CO_2_ for 15 min to allow initial EC attachment. Subsequently, 6 mL of HUVEC media was added to each well and maintained the constructs under static culture for 5 days to achieve full endothelialization. To form a uniform endothelium, cellularized constructs were flipped 180° once a day until incorporating them into the perfusion system at day 5.^[^ ^36^, ^43^ ^]^

### 2.10 Design and Assembly of Perfusion Systems with Magnets for 3D In Vitro Culture and NP Targeting

**Figure 1:**
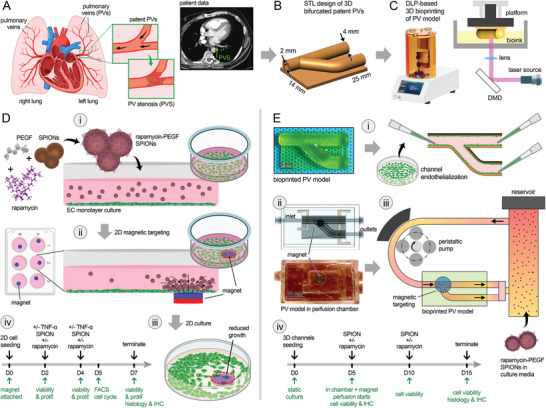
Schematic illustration of the experimental design used in this study to assess magnetic nanoparticle (NP) targeting of endothelium in bifurcated pulmonary vasculature. A) Schematic illustration of the human pulmonary vein (PV) structure in the healthy and stenotic states, i.e., PV stenosis (PVS). The inset on the rights shows the CT image of PVS (green arrow) in a human patient. B,C) A 3D standard tessellation language (STL) design of the bifurcated PVs was created, inspired by the anatomical patient data (A), and bioprinted using a digital light processing (DLP)‐based bioprinter (C). D) Prior to testing NP targeting in 3D PV models, 2D (monolayer) human umbilical vein endothelial cell (HUVEC) cultures were used to optimize and assess the impact of targeted delivery of drug‐loaded magnetic NPs on cell viability and proliferation. Rapamycin‐loaded superparamagnetic iron oxide NPs (SPIONs), coated with cross‐linked poly (ethylene glycol)‐co‐fumarate (PEGF) (i), were added to the EC culture media, while an N40 magnet was used externally to target the SPIONs within a randomly selected area within each well (ii). Cell cultures with SPIONs, with or without (control) rapamycin, were continued for 5 days and the reduced cell growth was analyzed via AlamarBlue, Live/Dead, and FACS cell cycle analyses (iii,iv). The timeline used for 2D cell culture assays is shown in (iv). E) 3D dynamic EC culture and NP targeting were conducted using the 3D bioprinted model of bifurcated PVs (A‐C). HUVECs were manually seeded onto the luminal space of printed channels (i), inserted within custom‐printed resin chambers (ii), and perfused via an 8‐channel peristaltic pump (iii). SPIONs ± rapamycin were added to the circulating media in the bioreactor, while an N40 magnet was inserted in a pocket inside the perfusion housing to target the SPIONs at the bifurcation site (iii). The bifurcation site was selected as the area at high risk of PVS. Dynamic 3D cultures started following a 5‐day static culture and continued for 10 days. Cell viability and growth were analyzed following the timeline shown in (iv).

A 3D perfusion chamber was designed on SOLIDWORKS (SolidWorks Corp., US) and 3D printed using a Form 3 stereolithography (SLA) printer (Formlabs, US) using a clear resin.^[^ ^27^, ^36^, ^43^ ^]^ The chamber consisted of one inlet and two outlets, allowing to direct the flow through the bifurcated vein structure. The chamber was designed to adequately house and fit the bioprinted PV constructs together with an N40 magnet in a cavity in the top lid of the perfusion chamber, directly above the bifurcation point (**Figure** **1**; Figure S1, Supporting Information). After printing, the perfusion chambers were washed (2 × 30 min) in 100% isopropanol to remove the residual non‐cross‐linked resin. The chambers were next dried and cured with UV light for 20 min. Subsequently, an N40 magnet was glued into the lid of the chamber with UV glue (Bondic, US) and aquarium sealant (Marineland, US) (Figure 1; Figure S1, Supporting Information). Silicon tubes and connectors were all sterilized by autoclaving prior to perfusion assays.

Following a 5‐day static culture (to achieve full endothelialization), bioprinted PV constructs were inserted into the perfusion housing. A 10% GelMA bioink solution was cast onto the constructs and UV cross‐linked (20 mw cm^−2^, 2 min) to ensure full coverage and sealing of the inlet and outlet interfaces. To seal the top and bottom chamber openings glass coverslips were mounted using UV glue and aquarium sealant, while allowing real‐time visual inspection and microscopy of the constructs. An 8‐channel peristaltic pump (Ismatec IPC digital peristaltic pump ISM933, US) generated the target flow rate of 2 mL min^−1^ through the vein‐like channels. Seven milliliters culture media was used to fill the reservoir and the tubing for each perfusion channel. Experimental groups included control (perfused with blank media without NPs), 3D constructs perfused with plain (no rapamycin) SPIONs, and constructs perfused with rapamycin‐loaded SPIONs. Following 10 days of dynamic culture, constructs were removed from the bioreactor, fixed in 10% formalin solution for 1 h, washed with PBS (3 × 30 min), and stored at 4 °C.

### 2.11 Calculation of Magnetic Flux Density in 3D Bioprinted PV Models

Magnetic flux (Φ) is defined as the total magnetic field passing through a given perpendicular unit area, and magnetic flux density (*B_z_*) is the amount of magnetic flux passing through a unit area of the material of choice as a function of *z* (distance from the magnet). Magnetic flux density was calculated using known parameters for the neodymium disc magnet (grade N40, Amazing Magnets), including the remanence (*B_r_*) of 1.3 Tesla (T), the magnet radius (*R*, 2.0 mm), and thickness (*D*, 2.4 mm). Thereupon, *B* was calculated for the specific magnet configuration within the perfusion chamber as follows (Figure S1, Supporting Information):

(5) $$B_{z}=\frac{B_{r}}{2}\left(\frac{D+z}{\sqrt{R^{2}+{\left(D+z\right)}^{2}}}-\frac{z}{\sqrt{R^{2}+z^{2}}}\right)$$

### 2.12 Simulating Endothelial Inflammation in 2D and 3D Cultures

To simulate the inflamed state of pulmonary vasculature in 2D and 3D models, an inflammatory cytokine, i.e., recombinant tumor necrosis‐α factor (TNF‐α) expressed in HEK 293 cells (Millipore Sigma, US), was used.^[^ ^44^, ^45^ ^]^ TNF‐α was added at a concentration of 10 ng mL^−1[^ ^46^ ^]^ starting at day 2 of static culture in 2D experiments and day 0 of dynamic culture for 3D experiments and maintained for the entire duration of culture.

### 2.13 In Silico Simulation of Flow in PV Constructs under Magnetic Flux

Computational fluid dynamics (CFD) modeling was used to predict the flow behavior in the PV constructs in the presence and absence of the magnetic flux. The CAD model of the bioprinted PV was used to create the geometry of the lumen walls. The intraluminal space, engulfing the fluid domain, was meshed in ANSYS ICEM using tetrahedral elements. The variation of magnetic flux density (*B*) within the 3D space across the intraluminal space was calculated using Equation (5) and specified as external body force in the fluid domain. The simulations were performed in ANSYS Fluent with the flow rate and pressure being prescribed at the inlet and outlet, respectively. The pulsatile inlet flow rate waveform was the same as that used in in vitro perfusion and the outlet pressure was set to be zero Pa (gauge). A developed parabolic velocity profile was assumed throughout the fluid domain. The fluid velocity at the inlet cross section was specified. Once the simulations were performed, the distribution of wall shear stress (WSS), velocity streamlines, dynamic pressure, and flow velocity in the *Z* direction (along the magnet flux) were obtained in post processing.

### 2.14 Cell Viability and Proliferation Assays

The non‐invasive AlamarBlue assay examined the cell viability and metabolic activity at serial time points throughout the 7‐day static culture of 2D samples (days 2, 4, and 7). Also, Live/Dead (L/D) assay was used to assess the viability of 2D static cultures (days 2, 4, and 7) and 3D dynamic cultures (days 0, 5, and 10 of flow).^[^ ^36^, ^47^ ^]^ In 2D static cultures, the AlamarBlue reagent (Bio‐Rad, US) was prepared at 10% v/v with fresh EC media and added to each well of the 12‐well culture plates. Cellular constructs were incubated in the dark for 3 h at 37 °C. Subsequently, 100 µL of the AlamarBlue mixture (*n* = 3) was collected from each well and transferred into a 96‐well plate to measure absorbances at 550 and 600 nm wavelengths using a microplate reader (Synergy 2, BioTek, US). AlamarBlue reduction percentage was calculated based on the previously established protocol, as a measure of cell viability and proliferation.^[^ ^47^, ^48^ ^]^

Terminal L/D assays on 2 and 3D PV cultures were conducted by aspirating the culture media from the wells of 12‐well plates (2D) or from the perfusion chambers of the 3D flow cultures and washing the samples with fresh EC media. The dye solution was prepared by mixing the Calcein‐AM and PI (Biotium, US) in EC media to obtain dilution ratios of 1:1000 and 1:50, respectively. Next, the culture media was aspirated and the staining solution was added to each well (2D) or to the construct (3D). Samples were incubated at 37 °C for 10 min (2D samples) or 45 min (3D samples). After the staining phase was completed, the dye was aspirated out and the samples were washed with fresh EC media. The samples were imaged using an epifluorescence microscope (Leica Microsystems DFC7000T, Germany). Cell viability percentage % was calculated by dividing the number of live cells (green signal) by the total number of cells (green + red signal).

### 2.15 Immunohistochemical Analysis

Following fixation and the subsequent washing, both 2 and 3D samples were prepared for immunohistochemical (IHC) analysis. 2D EC cultures were permeabilized by adding 0.5% Triton‐X 100 (Electron Microscopy Sciences, US) for 30 min and then blocked with 2% bovine serum albumin (BSA) (EMD Millipore, US) and 5% donkey serum solution (Sigma Aldrich, US) for 1 h. HUVEC monolayers were stained with CD31 (EC marker) and Ki‐67 and Phospho‐Histone H3 (PH3) to assess proliferation. Constructs were immunostained in constant motion using a primary antibody against CD31 (1:400, ab76533, Abcam, US), Ki‐67 (1:400, PIPA519462, Fischer Scientific, US), and PH3 (ab115127, Abcam, US) for 2 h at room temperature. Cells/constructs were then washed with PBS (3 × 1 h), incubated overnight with Alexa Fluor 594 donkey anti‐mouse (1:400) for Ki‐67 or PH3, and Alexa Fluor 488 donkey anti‐rabbit (1:400) for CD31 at 4 °C. Finally, the samples were counterstained with 4′,6‐diamidino‐2‐phenylindole (DAPI, blue) and imaged using both a confocal laser scanning microscope (FV1000, Olympus, Japan) and an epifluorescence microscope (Leica Microsystems DFC7000T, Germany). ImageJ software was used to quantify the fluorescent signals.

For staining the 3D PV construct, the fixed constructs were first embedded in 5% agarose, to preserve internal hollow structure, followed by slicing (200 µm) using a vibratome (VT1200S, Leica Biosystems, Germany). Slices were first permeabilized using 0.5% Triton‐X 100 (Electron Microscopy Sciences, US) for 30 min and blocked with 2% BSA (EMD Millipore, US) and 5% donkey serum solution in PBS for 1 h. Subsequently, construct slices were stained with PH3 and Ki‐67 to assess proliferation, and CD31 to examine EC morphology and health state. The samples were immunostained using a primary antibody against pH3 (1:200, ab76533, Abcam, US), Ki‐67 (1:200, PIPA519462, Fischer Scientific, US) at 4 °C overnight. Next, the slices were washed with PBS for (3 × 1 h) and incubated overnight with Alexa Fluor 594 donkey anti‐mouse (1:400) for Ki‐67 and Alexa Fluor 488 donkey anti‐rabbit (1:400) for CD31 at 4 °C. Samples were counterstained with DAPI (blue) and imaged via a confocal laser scanning microscope (FV1000, Olympus, Japan) and an epifluorescence microscope (Leica Microsystems, Germany). Fluorescent signal quantification was performed using ImageJ.

### 2.16 Prussian Blue Staining

To visualize the SPIONs in cell cultures, following fixation and washing, 2D samples and 3D construct slices were prepared for Prussian blue staining. Equal volumes of potassium ferrocyanide solution (Abcam, US) and hydrochloric acid solution (Abcam, US) were mixed, forming the iron staining solution. The solution was added to each well per slice, incubated for 3 min, and then washed (2X) with Milli‐Q water. Next, samples were stained in the dark with nuclear fast red solution for 5 min. After incubation, samples were rinsed with Milli‐Q water (4 × 5 min), dehydrated in 95% and 100% ethanol. Ethanol residues were removed from the wells and the samples were mounted for later visualization.^[^ ^49^ ^]^

### 2.17 Cell Cycle Analysis via Flow Cytometry

For 2D samples, on day 5 of culture, HUVECs from each well were passaged by trypsin, transferred into 15 mL conical tubes, and centrifuged (280 g, 3 min). The formed pellets were washed with PBS and fixed in 66% ethanol and stored at 4 °C for 2 h. Next, the cells were pelleted again, supernatant was decanted, cells were washed with PBS, and resuspended in 1X propidium iodide (PI)/RNAse staining solutions (Abcam, USA). PI was used as a nuclear and chromosome counterstain, to measure the DNA contents for cell cycle quantification. After incubation in the dark and on ice for 30 min, the samples were prepared for flow cytometry. This analysis was conducted on a Symphony A5 Cell Analyzer (BD Biosciences, US). Results were quantified on FlowJo v.10.8.1 (FlowJo LLC, US). Cell cycle analysis was performed using the *Dean‐Jen‐Fox* model, using the B 710_50‐A parameter for all the scans, for all the different experimental groups.

### 2.18 RNA Sequencing Analysis of Bioprinted PV Constructs and PVS Patient Tissue

Cellular constructs were removed from bioreactor housings at the end of perfusion culture and the proximal (bifurcation point) and distal (remote to bifurcation) regions were cut out using a scalpel (5 mm diameter) for downstream RNA sequencing analysis. Also, PV tissues obtained from a healthy donor and a PVS patient were cut into small pieces (≈2 mm diameter). Different cellular samples were placed in 1.5 mL microcentrifuge tubes for cell extraction and RNA isolation. Constructs were incubated in a 1 mL mixture of 10 mg mL^−1^ of Collagenase I/pepsin (Millipore Sigma, USA) in 1X TrypLE (Gibco, USA). The resultant digest was centrifuged at 500 rpm for 5 min to pellet the released cells. The supernatant was removed and the cell pellet was lysed with lysis buffer using the RNeasy Micro kit (QIAGEN, USA) for RNA extraction.

The bulk RNA isolated above was delivered to the Emory Integrated Genomics Core (EIGC) for further processing and RNA‐seq library generation. The cDNA from samples was generated using the SMART‐Seq v4 Ultra Low Input RNA Kit (Takara Bio, USA). The final sequencing library was made using the Nextera XT kit (Illumina, USA). Quality control of samples was carried out using established protocols at the EIGC facility. Library sequencing was conducted on a Novaseq S4 flow cell (Illumina, USA) with yields of ≈100 m reads per sample. Library analysis, gene expression comparisons, and data visualization were performed using the ROSALIND platform (Rosalind.bio, USA).

### 2.19 Scanning Electron Microscopy (SEM) and Energy‐Dispersive X‐Ray Spectroscopy (EDS) of PV Constructs

Endothelialized 3D printed PV constructs were fixed as described above at day 10 post‐perfusion and dehydrated for SEM and EDS analyses. Briefly, each construct was cut in half using a microtome blade and then each half was incubated at room temperature in increasing concentrations of anhydrous ethanol in 1X PBS to dehydrate the samples. Concentrations of ethanol (0, 25, 50, 75, and 100%) in PBS were used, immersing for 24 h in each solution on a rocker plate. After 24 h in 100% ethanol, the liquid was aspirated off the sample, and the sectioned PV constructs were left to dry completely for another 24 h. Prepared samples were imaged on an AxiaChemiSEM (ThermoFisher) under high vacuum at 3 kV with magnification ranging from 150X to 3600X. Additionally, EDS analysis was performed in various regions within bioprinted PV geometries for samples treated with NPs and without NP (control) under the same conditions as the SEM imaging, at 900X magnification and with a 4 min detection window (*n* = 3).

### 2.20 Statistical Analysis

Data obtained from the AlamarBlue, L/D, IHC analyses, and flow cytometry measurements were presented as mean ± standard error of mean (SEM). Significant differences were determined with one‐way or two‐way ANOVA (GraphPad Prism). For the experiment consisting of only two groups, a student's t‐test was performed (GraphPad Prism). P values < 0.05 were considered statistically significant (*: *P* < 0.05, **: *P* < 0.01, ***: *P* < 0.001, and ****: *P* < 0.0001). A post‐hoc Tukey‐Kramer correction was performed for assays that required multiple parallel comparisons. A sample size of at least *n* = 3 was utilized for each statistical analysis.

## 3 Results and Discussion

Treatment options for PVS, primarily surgical and catheter‐based methods, have not provided satisfactory results and the pathophysiology of the disease is yet poorly understood. Recent studies have identified unique histopathological changes in the intimal hyperplasia of stenotic PV, including the EndMT driven by transforming growth factor‐b1 (TGF‐b1) expression, and the excessive proliferation of myofibroblast‐like cells in the lesion. This neoproliferative condition in PVS has motivated chemotherapy trials to inhibit the myofibroblastic growth. While these trials, particularly the combination chemotherapies (e.g., with imatinib and sirolimus), have stabilized the disease and improved short‐term survival, they have shown modest success in long‐term survival and treatment of these patients. Restenosis and cytotoxic side effects are among major complications caused by the systemic or stent‐assisted delivery of chemotherapies in PVS patients.^[^ ^20^, ^22^, ^23^ ^]^ To address these challenges, in this study, a 3D bioprinted in vitro model of bifurcated PVs was fabricated, cellularized, and perfused to enable examination of the cellular mechanisms underlying the (re)stenosis and the response to chemotherapies. Further, using drug‐loaded magnetic NPs (SPIONs) and an external magnetic field, a targeted delivery approach was evaluated as a new and more effective therapeutic regimen to treat PV stenosis and restenosis (Figure 1).

**Figure 2:**
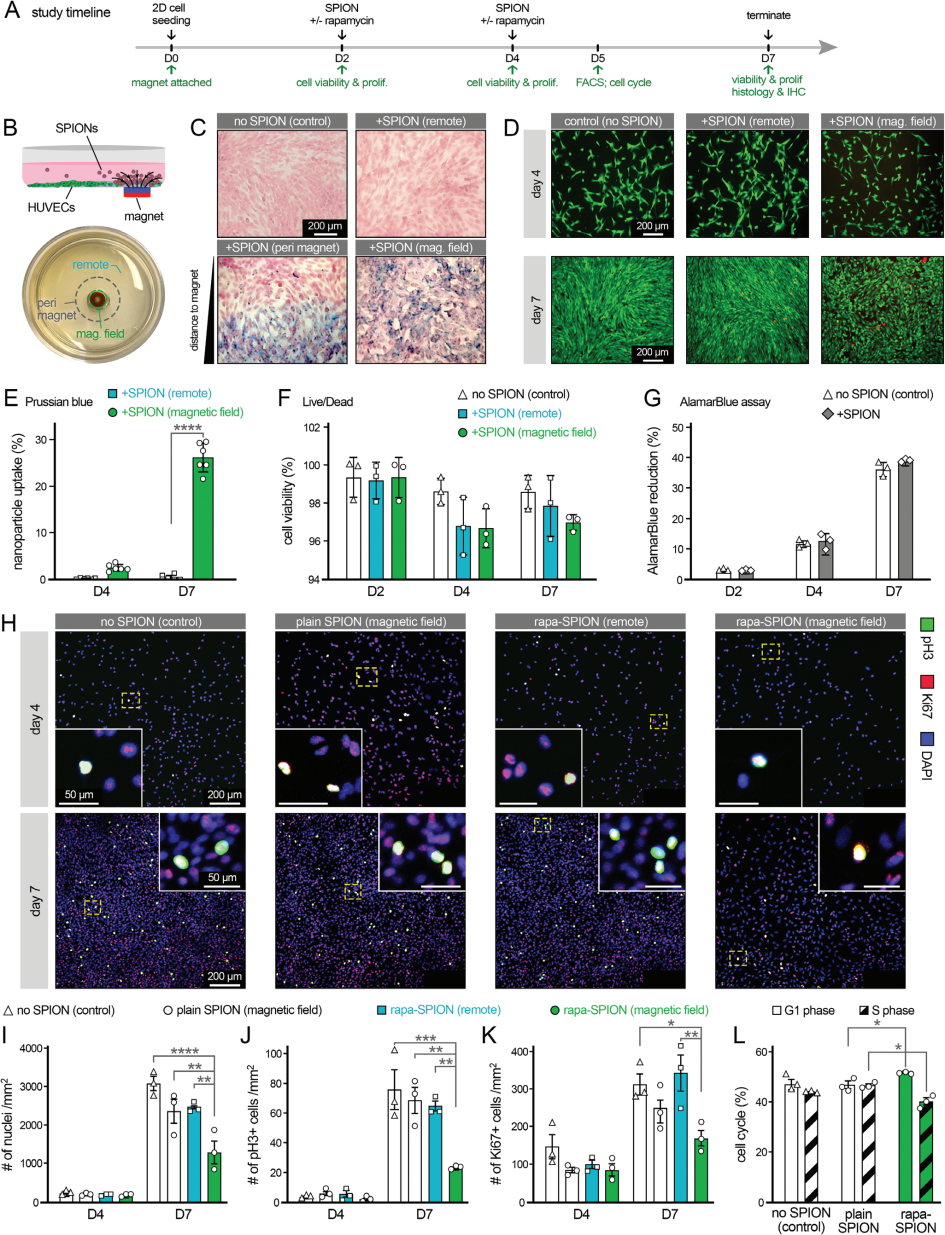
Studying the effect of targeted drug delivery of therapeutic nanoparticles on endothelial cells (ECs) in 2D culture. A) The study timeline and readouts used for 2D cell culture assays. B) Schematic (top) and photo (bottom) of a culture well, illustrating the magnetic targeting of rapamycin‐loaded superparamagnetic iron oxide NPs (SPIONs). Three main regions were defined in 2D cultures as magnetic field, peri magnet, and remote regions. C) Prussian blue staining used to detect iron in the EC cultures. Results are shown for control (without SPIONs), remote (far from the magnet), peri magnet (at the border zone of the magnet), and magnetic field (within the magnet region). D) Live/Dead assay results conducted at days 4 and 7 of 2D EC culture (red: dead cells, green: live cells). Scale bars in C and D show 200 µm. E,F) Quantification of the results obtained from Prussian blue (C) (*n* = 6) and Live/Dead (D) (*n* = 3) assays, respectively. No significant differences were obtained in Live/Dead measurements (F). G) AlamarBlue reduction percentage as a measure of cell viability and proliferation, obtained for control EC culture (without SPION) and cultures treated with drug‐loaded SPIONs. H) Immunostaining of ECs at days 4 and 7 of culture, treated with no SPIONS (control), plain SPIONs (no rapamycin loaded), and rapamycin loaded SPIONs from both remote and magnetic field regions. Blue stains represent nuclei (DAPI), and green and red stains highlight proliferation ECs (pH3 and Ki67, respectively). Insets show magnified views of proliferating cells in each group. Scale bars represent 200 and 50 µm. I–K) Quantification of immunostained groups for DAPI+ (I), pH3+ (J), and Ki67+ (K) cells, normalized by the surface area (mm^2^) (*n* = 3 per group). L) Cell cycle analysis results via fluorescence‐activated cell sorting conducted at day 5 of EC culture, indicating the percentage of cells in the G1 and S phases (*n* = 3 per group). *p < 0.05, **p < 0.01, ***p < 0.001, and ****p < 0.0001.

Magnetic drug targeting is extensively explored as an alternative drug delivery approach which, compared to the systemic delivery methods, significantly reduces the drug load and associated adverse side effects, hence maximizing the effect of drug at the target site.^[^ ^50^ ^]^ Various magnetic NP systems have been used for targeted drug delivery applications, particularly for the enhanced delivery of chemotherapeutic reagents in oncology.^[^ ^51^, ^52^, ^53^ ^]^ Among various magnetic NP‐based delivery vehicles, SPIONs have been increasingly used in biomedical applications, owing to their unique physicochemical and biological characteristics.^[^ ^54^, ^55^, ^56^, ^57^ ^]^ In this study, to evaluate the efficacy of magnetic targeting of rapamycin, loaded onto SPIONs, to inhibit EC growth, we first conducted a series of 2D cell culture assays (Figures 1D and **2**). Iron (Prussian blue) staining of monolayer HUVECs cultured with SPIONs (without drug) demonstrated effective accumulation of NPs within the magnetic flux density region generated by the external magnet positioned under each well (Figure 2A–C).^[^ ^54^ ^]^ The SPIONs concentrated within the magnet area were effectively uptaken by the HUVECs, while the remote and control culture (no SPIONs) showed no positive staining (Figure 2C). Of note, an evident NP distribution gradient was observed in the peri magnet field. The SPION uptake gradually decreased from the center toward the outside (remote) area, becoming undetectable at ≈3 mm radius from the magnet field's center (Figure 2C, arrow). L/D assay results demonstrated the cytocompatibility of SPIONs (without drug) in both magnet and non‐magnet zones (Figure 2D). Quantification of Prussian blue staining showed significantly (*p* < 0.0001) higher uptake of SPIONs in the magnet versus remote zones at day 7 of culture, following 5 days of particle treatment (Figure 2E). A remarkable 60X increase in SPION uptake was obtained in magnet versus remote regions on day 7. Quantification of L/D assays results showed high cell viability in all groups, exceeding 96% after 7 days of culture (Figure 2F). AlamarBlue assay results further confirmed HUVEC viability and growth, with no significant differences (*p* > 0.05) in SPION treated and untreated (control) cultures (without rapamycin) (Figure 2G).

To examine the effect of targeted delivery of rapamycin‐loaded SPIONs (rapa‐SPION) on ECs, monolayer HUVEC cultures were immunostained at day 7 of culture (5 days of NP treatment). Rapa‐SPION treatment yielded a significantly reduced number of cells (# of nuclei per surface area) within the magnet field, compared to the remote region of same cultures (48% reduction), compared to the plain SPION group (no rapamycin) (45% reduction), and compared to control cultures without SPIONs (58% reduction) (Figure 2H,I). PH3 and Ki67 staining and quantification indicated consistent reduction trends in the EC proliferation among various study groups, with the cells in the magnetic field of rapa‐SPION group exhibiting the lowest level of cell proliferation (Figure 2H,J,K). Compared to other groups, targeted rapa‐SPION treated HUVECs showed a 65–70% reduction in pH3 signal, and 32–51% reduction in the Ki67 signal. All experimental groups showed relatively lower nuclei counts as well as pH3 and Ki67 signals at day 4, compared to day 7, with no significant differences among the groups (*p* > 0.05). Significantly lower levels of CD31 signal were also measured from the magnetic fields treated with rapa‐SPIONs on both days 4 and 7 of culture (Figure S1A–C, Supporting Information). Cell cycle analysis via FACS indicated that HUVECs treatment with rapa‐SPIONs resulted in a significant (*p* < 0.05) increase in the percentage of cells that are likely to be arrested at the G1 phase and a decrease in percentage of cells in the S phase, in comparison to plain NPs (Figure 2L). Together, these results indicate the efficacy of external magnetic field in targeted delivery of rapamycin loaded NPs, and their effective function to inhibit vascular cell growth in 2D cultures.^[^ ^18^, ^19^, ^20^ ^]^

Once we validated the feasibility and efficacy of targeted rapamycin delivery to inhibit EC growth in 2D cultures, we aimed to examine this effect under pathological conditions that more closely recapitulate the PVS. Elevated levels of various potent proinflammatory factors, such as TNF‐α, have been reported in different types of vascular disease.^[^ ^58^ ^]^ TNF‐α is a key inflammatory cytokine that activates and regulates the growth of vascular cells and its pivotal role in disrupting endothelial structure and function both in vivo and in vitro has been established.^[^ ^59^, ^60^, ^61^ ^]^ Addition of TNF‐α (10 ng mL^−1^) to the culture media for five days resulted in significant (*p* < 0.05) increase in HUVEC proliferation in both control (no SPIONs) and rapa‐SPION culture groups (Figure S1D,E, Supporting Information). Treatment with this proinflammatory factor also decreased the percentage of cells in the G1 phase, confirming its pro‐proliferative effect on HUVECs (Figure S1F, Supporting Information). Of significance, the rapa‐SPION treatment was still effective in diminishing cell growth under the pathological condition with elevated TNF‐α level. These results confirm the efficacy of targeted therapy approach to address pulmonary vascular disease such as PVS.

Following the 2D examination of the effect of targeted delivery of rapa‐SPION on EC behavior, we conducted 3D cell culture assays to further enhance the biomimicry and clinical relevance of our study. For this purpose, we employed an advanced DLP‐based bioprinting method and a novel formulation of GelMA‐based bioink (developed by our group) to create the 3D in vitro models of bifurcated PV (Figure 1B,C,E). To date, a variety of 3D bioprinting modalities are increasingly used to create 3D scaffold structures recapitulating the native tissue structure and/or function.^[^ ^62^, ^63^, ^64^ ^]^ Among various techniques, DLP‐based bioprinting has shown great promise in fabricating complex 3D geometries at relatively high speed and precision.^[^ ^65^ ^]^ The bioink developed in this study to print the vascular models consisted of porcine GelMA, cold water fish gelatin, and PEGDA6000 hydrogels, yielding enhanced cell viability, while maintaining printability of large, anatomically relevant structures via the DLP bioprinting modality. This bioink formulation was also robust enough to allow for relatively high flow rates that recap the physiologic environment in vivo over the long periods of cultures. While these features have been partially established in prior reports,^[^ ^31^, ^42^, ^43^, ^65^, ^66^, ^67^, ^68^ ^]^ the developed bioink formulation here fulfilled all these key biomanufacturing requirements. Therefore, the modified ink formulation offers promise for future attempts at bioprinting of more complex, 3D cell‐encapsulated tissue geometries that can provide long‐term cell‐cell, cell‐ECM, and cell‐microenvironment interactions.^[^ ^68^, ^69^ ^]^

**Figure 3:**
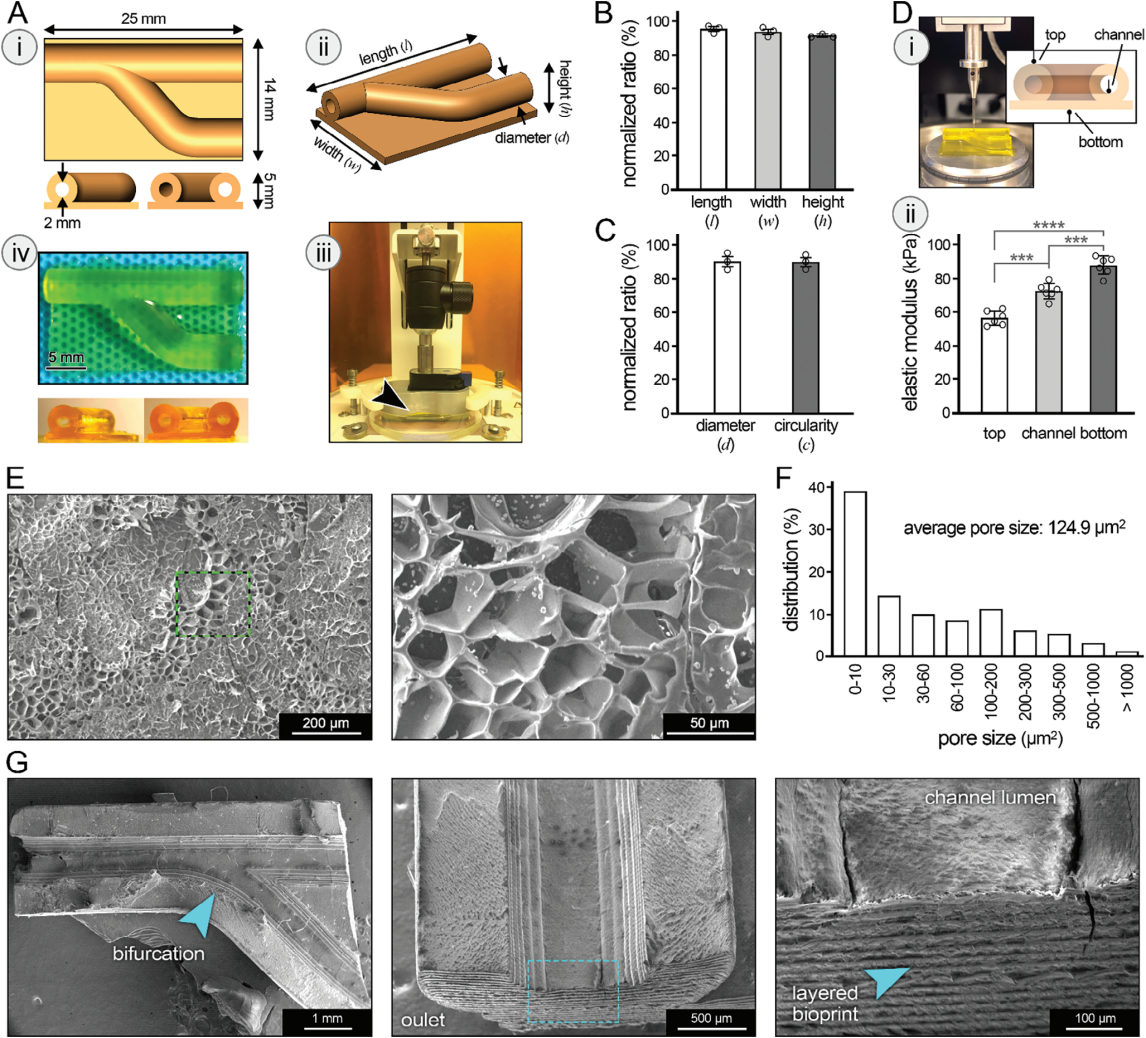
Characterization of 3D bioprinting fidelity, mechanical properties, and pore structure for fabricated pulmonary vein (PV) models. A) Computer aided design (CAD) of the bifurcated PV geometry (i,ii) was generated, inspired by the clinical data, and printed using a digital light processing (DLP) bioprinter (iii), resulting in the fabrication of gelatin methacrylate (GelMA)‐based hydrogel constructs with one inlet and two outlets (iv). Bottom insets in (i) and (iv) are the side views, showing the inlet and outlets. The scale bar represents 5 mm. B,C) Printing fidelity of fabricated PV models were quantified by measuring various structural parameters and normalizing them by the corresponding parameter in the CAD model (*n* = 3). Parameters included the length (*l*), width (*w*), and height (*h*) of 3D constructs (B), as well as the channel diameter (*d*) and their circularity (*c*) (C). D) Microindentation of bioprinted models (i) was used to quantify the elastic modulus of constructs at different areas (ii), including the top and bottom of constructs, as well as the luminal surface in the channels (*n* = 6 per group). E,F) Scanning electron microscopy (SEM) of bioprinted PV constructs was used to assess the pore structure (E), which was further quantified in the pore size distribution histogram (*n *= 4) (F). An average pore size of 124.9 µm^2^ was measured. G) SEM imaging also revealed the surface topography and layered structure of bioprinted vasculature. *p < 0.05, **p < 0.01, ***p < 0.001, and ****p < 0.0001.

Some of the key aspects that would quantitatively define the quality (precision and reproducibility) of bioprinted constructs are the bioprintability (i.e., printing fidelity) and mechanical properties of printed scaffolds.^[^ ^31^, ^34^, ^67^ ^]^ Structural fidelity measurements on printed PV constructs demonstrated adequate levels of precision (>90% accuracy compared to the CAD model) for the length (*l*), width (*w*), and height (*h*) of the constructs, as well as the printed channels diameter (*d*) and their circularity (*c*) (**Figure** **3**A–C). This level of DLP bioprinting fidelity is consistent with the levels previously reported by us^[^ ^30^, ^31^, ^34^, ^67^ ^]^ as well as other groups.^[^ ^65^, ^70^, ^71^ ^]^

Elastic moduli of bioprinted PV model obtained from the top and bottom surfaces and the channel lumen surface were significantly different (*p* < 0.001, 0.0001), measured at 56.4 ± 4.3, 72.2 ± 4.7, and 87.6 ± 5.4 kPa, respectively (Figure 3D). The increase in stiffness from top to bottom surface of bioprints could be attributed to partial consolidation of soft hydrogel due to gravity,^[^ ^47^, ^72^ ^]^ as well as the light penetration and over‐cross‐linking of the initial layers of constructs, which is inherent to the DLP bioprinter used in this study (LumenX).^[^ ^26^, ^73^, ^74^ ^]^ The observed mechanical gradient within the printed model highlights the important capability of microindentation, compared to bulk mechanical testing methods, to probe the heterogeneous mechanical patterns within 3D structures.^[^ ^75^ ^]^ Of note, the range of elastic modulus measured for printed PV models was comparable to that reported for native PV tissues.^[^ ^76^, ^77^, ^78^ ^]^

SEM images of printed PV constructs revealed the highly porous structure of GelMA‐based scaffolds (Figure 3E,F). Over 80% of pores were smaller than 200 µm^2^ in surface area (≈8.0 µm in diameter, assuming a circular pore shape). An average pore area of 124.9 µm^2^ (≈6.3 µm in diameter) and total porosity of 37.86% were measured based on the SEM analysis (Figure 3F), indicating the sufficient capacity of bioprinted scaffolds to support the exchange (diffusion) of oxygen and nutrient molecules, as well as various NP systems (e.g., SPIONs used in this study).^[^ ^79^, ^80^ ^]^ SEM imaging also revealed the jagged topography of PV lumen surfaces, which is an inevitable structural feature generated by the layer‐by‐layer DLP bioprinting process (Figure 3G).

Strong superparamagnetic properties of SPIONs, together with their adequate biodegradability, biocompatibility, and efficient clearance from the body (via iron metabolism pathways), have made these particles a highly attractive nanobiomaterial in a plethora of biomedical applications.^[^ ^55^, ^81^, ^82^ ^]^ Specifically, their strong magnetic properties have enabled them to serve as robust diagnostic agents in magnetic resonance imaging (MRI),^[^ ^83^ ^]^ as well as nano‐transporters in a variety of targeted drug delivery applications, where the drug‐loaded SPIONs can be guided precisely to the target site via an external magnetic field.^[^ ^55^, ^84^, ^85^ ^]^ Various surface modifications of SPIONs with drugs along with the application external magnetic field have resulted in enhanced delivery and therapeutic efficacy in diseases such as cancer (e.g., paclitaxel loaded SPIONs for fibrosarcoma therapy),^[^ ^86^ ^]^ cardiovascular disease (e.g., SPIONs uptaken by endothelial progenitor cells and their targeted delivery to treat injured carotid artery),^[^ ^87^ ^]^ and central nervous system disorders (e.g., using SPIONs to bypass blood‐brain barrier).^[^ ^88^ ^]^ For drug delivery applications, SPIONs are typically coated with hydrophilic and biocompatible polymers to form an iron oxide core and a hydrophilic surface structure, where drugs can be loaded in the coating layer.^[^ ^89^ ^]^ Following our established protocol,^[^ ^90^, ^91^ ^]^ the rapamycin‐loaded cross‐linked PEGF‐SPIONs were successfully created for this study. DLS measurements showed an average hydrodynamic size of 25.1 nm ± 4.4 (PDI: 0.26) and 78.9 nm ± 21.2 (PDI: 0.39) for the PEGF‐coated SPIONs before and after rapamycin loading, respectively. TEM analysis of the SPIONs confirmed the efficient drug loading onto the particles, and relatively uniform size distribution.

**Figure 4:**
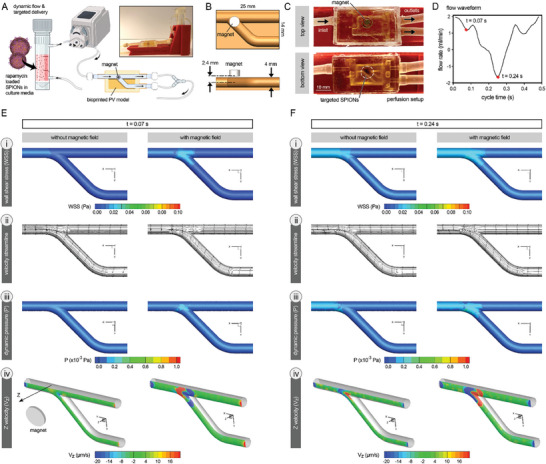
Experimental and computational platforms used for dynamic culture of bioprinted pulmonary vein (PV) models and their magnetic nanoparticle (NP) targeting. A) Experimental set‐up designed for perfusion culture of bioprinted PV models, allowing for circulation of superparamagnetic iron oxide NPs (SPIONs), loaded with rapamycin drug, and their targeted accumulation at the bifurcation site. B,C) Configuration of magnet within the perfusion chamber design to allow for effective NP targeting at the bifurcation site. The bottom panel in (C) highlights the accumulation of SPIONs at the target site, i.e., the bifurcation site (white circle). D) The flow waveform utilized in this study to perfuse PV constructs using a peristaltic pump. Two time points within one 0.48 s cycle were used in computational fluid dynamics (CFD) modeling (E‐F) to examine flow hemodynamics. E,F) Prediction of flow hemodynamics parameters via CFD for *t* = 0.07 s (E) and *t* = 0.24  (F) within one cycle of pulsatile flow. The examined parameters included wall shear stress (WSS, Pa) i), velocity streamlines ii), dynamic pressure (P, x 10^−3^ Pa) iii), and Z velocity (V_z_, µm s^−1^, toward the magnet) (iv).

Following the in‐depth characterization of bioprinted PV models, and analyzing drug‐loaded SPIONs, we conducted 3D assays of targeted drug delivery under dynamic flow conditions (**Figure** **4**A–C). For this purpose, a flow rate of 2 mL min^−1^ was employed which was noticeably smaller than that in the native human PV tissues (3–4 L min^−1[^ ^92^ ^]^). The lower flow rate was selected due to the limitations in perfusing higher flow rate which could cause significant damages to the soft bioprinted tissue and pose risks of leakages and/or hydrogel degradation.^[^ ^36^, ^42^ ^]^ Further, the inner diameter of bioprinted channels (2 mm) was significantly smaller than those reported for native human PVs (Figure 4A,B), ranging from 9 to 13 mm.^[^ ^93^ ^]^ The scale‐down in the in vitro models, together with the mismatch in viscosity of blood versus water (culture media) used in vitro, should be considered to accurately calculate the prescribed flow rate (as applied in our prior works^[^ ^36^, ^43^ ^]^). While this study serves as proof of principle, future works could attempt at applying more physiologically relevant flow rates to better recapitulate the flow hemodynamics parameters, such as wall shear stress (WSS), that are present in the native PVs.^[^ ^94^, ^95^ ^]^

Flow disturbances at vascular regions near bifurcations and curvatures are reported to result in complex spatiotemporal hemodynamic patterns (e.g., drastically altered WSS).^[^ ^96^, ^97^, ^98^ ^]^ Identifying such alterations can predict the susceptibility of vascular tissues to various abnormalities, including stenotic and atherosclerotic lesions.^[^ ^97^, ^98^ ^]^ In this study, we used CFD modeling of flow in the bioprinted PV geometry to predict the distribution of WSS (Pa), velocity streamlines, dynamic pressure (*P*, Pa), and flow velocity in the *Z* direction (toward the magnet) (*V_z_*, µm s^−1^) within the 3D channel spaces (Figure 4D–F; and Videos S1–S12, Supporting Information). Results showed altered flow hemodynamic patterns particularly at the bifurcation point. The altered and disturbed flow patterns were amplified at *t* = 0.24 s which corresponded to the maximum negative flow rate within the construct (Figure 4F). According to these data, the ECs lining the printed channels lumen at the bifurcation region are exposed to the highest levels of alterations in flow hemodynamics, and therefore, are prone to dysregulation in their behavior, including the cellular overgrowth.^[^ ^99^ ^]^

To inhibit the abnormal EC growth, we circulated rapamycin‐loaded SPIONs in the culture media and utilized a magnet, inserted within the perfusion chamber set‐up (Figure 4A–C), to enable targeting of drug‐loaded NPs at the bifurcation site. Using Equation 5 (see Experimental Section) we calculated the magnetic flux density, *B_z_* = 0.4 T at a distance from the magnet, *z* = 0.4 mm, which is the gap between the magnet surface and the top channel lumen surface (Figure S2, Supporting Information). The magnetic flux density calculated for our geometry was consistent with prior reports (0.5 – 2 T) utilizing similar magnet types for NP targeting applications.^[^ ^100^ ^]^ Computation modeling of *B_z_* distribution in the x‐y plane at the top lumen surface of channels (*z*  =  0.4 *mm*), also confirmed the 0.4 T magnetic flux at the center of the magnet, which was accurately focused onto the vascular bifurcation zone (Figure S2C, Supporting Information). Modeling of *B_z_* in the z direction (toward the magnet), demonstrated a rather uniform magnetic field flux distribution across the lumen (Figure S2D, Supporting Information). Magnetic field attenuation due to circulating medium was deemed negligible.^[^ ^101^ ^]^ Incorporation of magnetic traction force into the CFD modeling resulted in further (modest) increase in the alterations in WSS (0.01 to 0.02 Pa), *P* (0.0001 to 0.0002 Pa), and *V_z_* (−20 to 16 µm s^−1^) at the bifurcation site for both *t* = 0.07 and 0.24 s (Figure 4E,F). Although the velocity streamlines in the flow direction were slightly perturbed by the presence of the magnetic field, the more significant alteration in the velocity occurred along the magnetic flux, normal to the flow direction. This could explain the modest effect of the magnetic field on the WSS along the flow direction. Therefore, CFD simulations serve as valuable means to predict and modulate the additional potential disturbances in the flow due to the presence of a magnetic field. In addition, the bifurcation region often causes the formation of vortices when the flow reverses (Videos S2 and S8, Supporting Information). The presence of vortices leads to brief periods of oscillatory flows which could act as another independent mechanism leading to EC dysregulation.^[^ ^102^, ^103^ ^]^

These data suggest the effective role of external magnetic field in accumulating the SPIONs at the target site (Videos S4,S5,S10, and S11, Supporting Information), as was also evident in the bottom view images of the perfusion constructs (Figure 4C, bottom). It should be noted that the slight alterations in WSS (0.01–0.02 Pa), induced by the exertion of magnetic force,^[^ ^101^, ^104^ ^]^ may also have a contribution to the EC behavior in the targeted regions and could be more carefully examined in future studies.^[^ ^105^ ^]^

**Figure 5:**
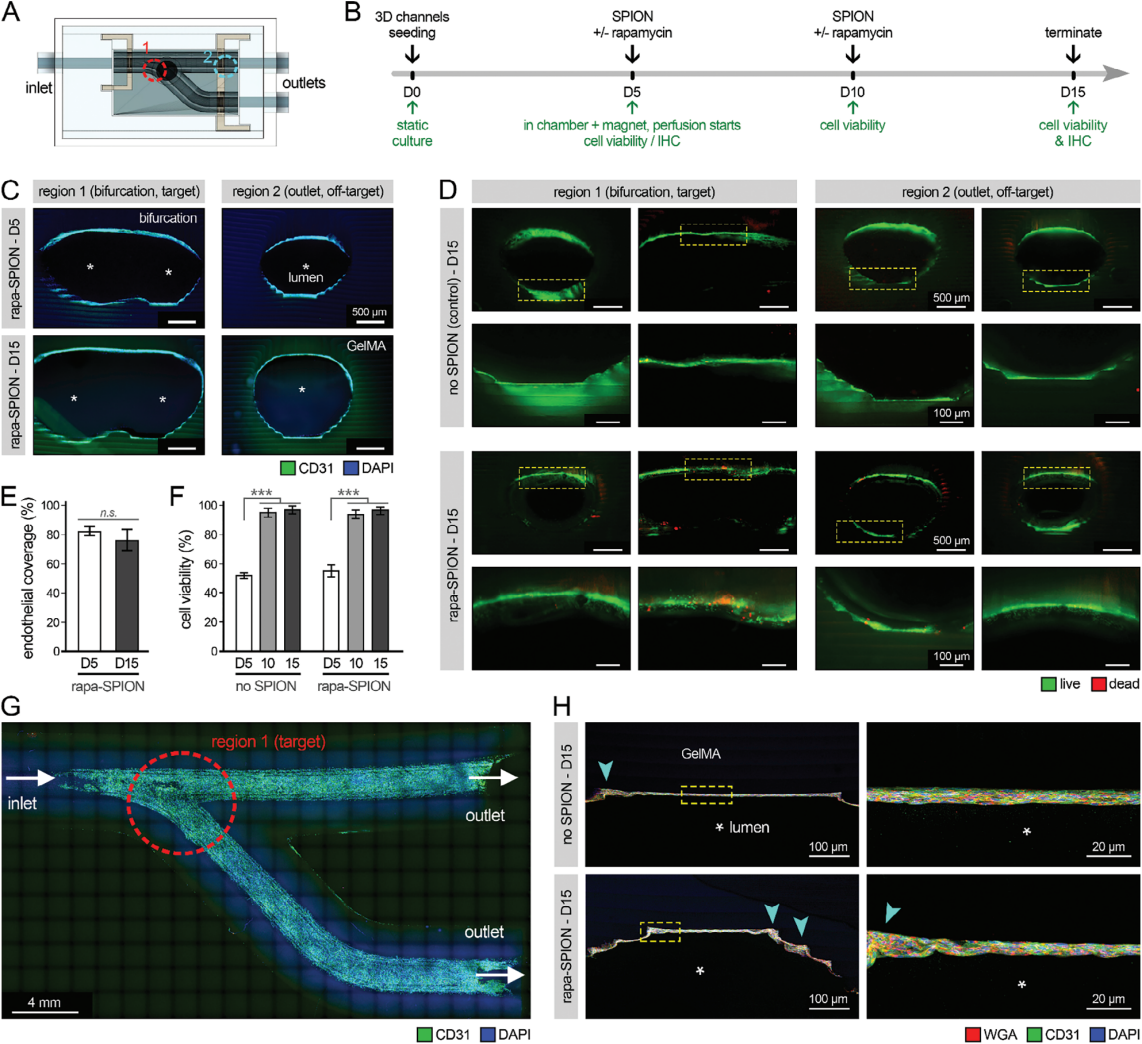
Assessment of endothelialization on the lumen surfaces of bioprinted vascular constructs under dynamic culture. A) Schematic diagram of the perfusion chamber design, housing the bioprinted pulmonary vein (PV) constructs. Regions 1 (red) and 2 (sky blue) were selected to represent the target (bifurcation point) and off‐target (outlet) regions, respectively, that were used in this study to assess drug targeting. B) The overall study timeline for 3D static‐dynamic culture assays. C) Immunohistochemical (IHC) imaging of PV channels in regions 1 and 2 (target and off‐target), seeded with human umbilical vein ECs (HUVECs) and treated with rapamycin‐loaded superparamagnetic iron oxide nanoparticles (rapa‐SPIONs) at days 5 (end of the static culture) and 15 (end of dynamic culture). The stars highlight the lumen space. Scale bars indicate 500 µm. D) Live/Dead assay conducted on day 15 of culture of PV constructs, in regions 1 and 2, treated with no SPIONs (control, top row) and with rapa‐SPIONs (bottom row). Green shows live cells and red highlights dead cells. Bottom row for each group shows the magnified view of the region highlighted (yellow box) in the top row. Scale bars in top and bottom rows of each group indicate 500 and 100 µm, respectively. E,F) Quantification of endothelial coverage (E) and HUVEC viability (F) in various groups during days 5 to 15 of culture (obtained from IHC images in C‐D). G,H) IHC imaging of bifurcation region (1) in PV construct treated with rapa‐SPION (G) and with and without rapa‐SPION (H) at day 15 of culture. Images on the right show magnified views of the thin endothelium at the bifurcation region (yellow boxes). The cyan arrows point at the jagged surface features on the surface of printed channels. Constructs were stained for WGA (red), CD31 (green), and DAPI (blue). *p < 0.05, **p < 0.01, ***p < 0.001, and ****p < 0.0001.

Following 10 days of perfusion, with and without SPIONs, we stopped the dynamic cultures and examined the endothelialized PV constructs to assess the NP targeting at the bifurcation site and its impact on the endothelium. The EDS results confirmed effective accumulation of SPIONs at the bifurcation region (#1) within the PV constructs, while the off‐target region (#2) showed noticeably lower levels of Fe peak intensity (Figure S3, Supporting Information). IHC imaging of PV constructs at the beginning (day 5) and end (day 15) of dynamic culture demonstrated high levels of channel endothelialization and HUVEC viability in both target (#1) and off‐target (#2) regions within the 3D constructs (**Figure** **5**C,D). Quantification of IHC images for DAPI and GFP signal indicated a >76% luminal coverage (of the total area) by the HUVECs in the rapa‐SPION treated constructs, while there were no significant differences (*P* > 0.05) between target and off‐target regions, and between the day 5 and 15 results (Figure 5C,E). The accuracy of EC coverage quantification could, however, be impacted by the staining artefacts from the GelMA and the construct edge. Quantified Live/Dead data also showed significant increases (*P* < 0.001) in cell viability following the start of dynamic flow, from day 5 to days 10 and 15, for both control (no SPIONs) and rapa‐SPION groups (from 55.5% on day 5 to 94.6% and 97.4% on days 10 and 15, respectively) (Figure 5F). No significant adverse effect was observed on EC viability for the rapa‐SPION treatment compared to the control (97.4 ± 1.0% in drug treated group versus 97.6 ± 1.0% in control). The increased EC viability could be attributed to enhanced diffusion and accessibility of nutrients and oxygen for the cells within 3D constructs,^[^ ^36^, ^43^ ^]^ as well as the endothelial cell remodeling and mechanotransduction signaling triggered by the flow‐induced wall shear stress on the lumen surface^[^ ^102^, ^106^ ^]^ (Figure 4). The whole mount imaging (horizontal section) of PV constructs confirmed the complete and uniform endothelialization of channels under flow (Figure 5G). Further IHC examination of rapa‐SPION and control groups in the target (#1) region, demonstrated a thin healthy layer of HUVECs, consisting of 2–3 cell layer), expressing uniform CD31 and WGA signal after perfusion for 10 days (Figure 5G,H). The layered structure and jagged surface topography of printed lumens were evident in higher magnification images, which are inherent to the DLP‐based bioprinting technique used to create these constructs. Together, these results confirmed the capability of bioprinted GelMA based constructs to form robust endothelium under the dynamic flow and showed no adverse toxicity effects from SPIONs circulated through the PV channels.

**Figure 6:**
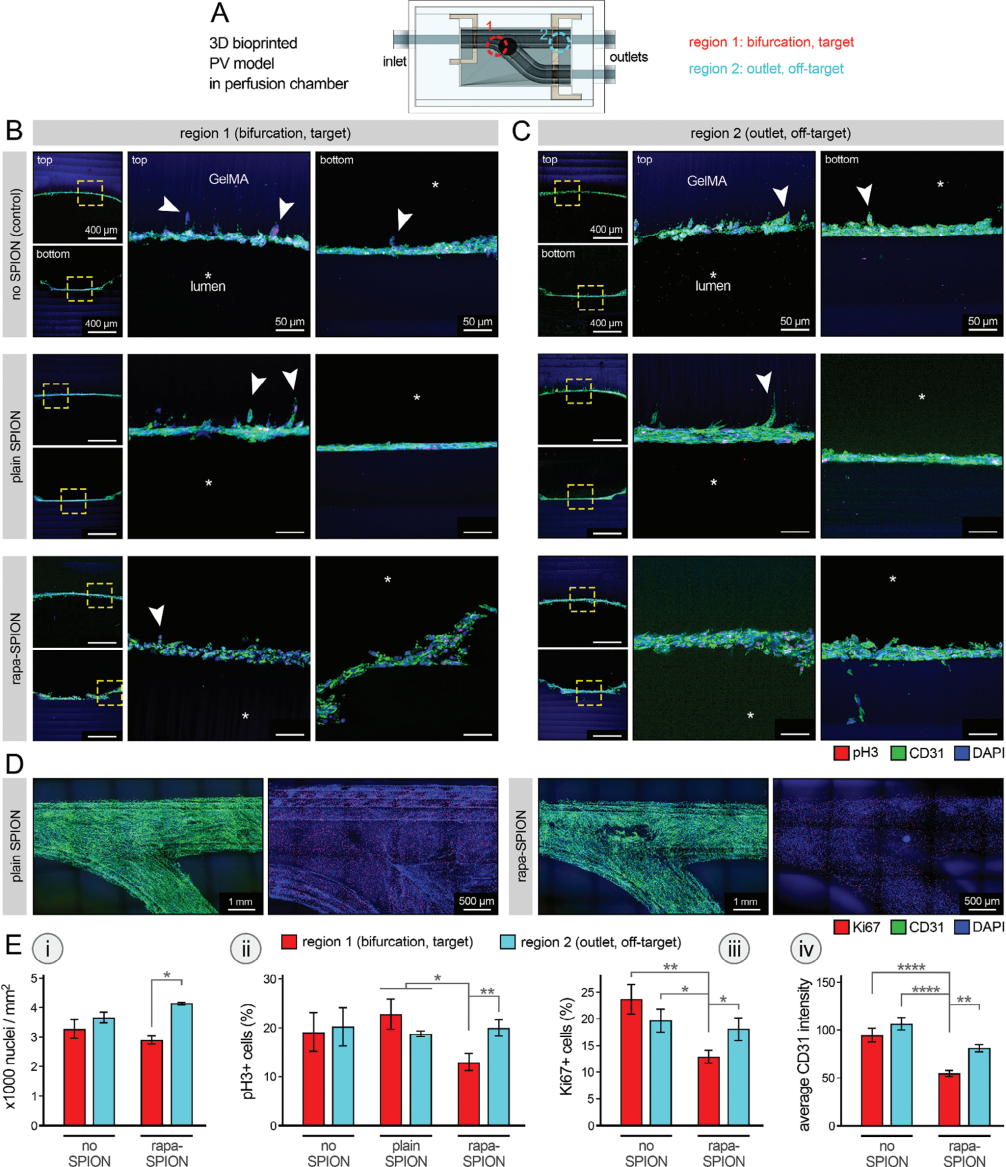
Evaluating the endothelial cell (EC) response to targeted delivery of therapeutic nanoparticles (NPs) in 3D bioprinted pulmonary vein (PV) platform. A) Schematic diagram illustrating the main regions 1 (bifurcation point, target) and 2 (remote/outlet area, off‐target) that were examined in this study. B,C) Immunohistochemical (IHC) analysis of endothelialized PV constructs cultured under dynamic flow conditions without superparamagnetic iron oxide NPs (SPIONs) as control (top row), and those perfused with plain SPIONs (no drug, middle row) and rapamycin‐loaded SPIONs (rapa‐SPION, bottom row). IHC images of human umbilical vein ECs (HUVECs), stained for pH3 (red), CD31 (green), and DAPI (blue) are shown for region 1 (target, B) and region 2 (off‐target, C). Yellow boxes in the panels on the left highlight the zones at magnified views on the right (tom and bottom of the vessel channel). White arrows in B‐C point to the EC alignment perpendicular to the channel axis. Scale bars represent 400 and 50 µm. D) IHC images of HUVECs in bifurcation zone (region 1) of PV constructs, treated with plain SPION (left, control) versus rapa‐SPION (right), stained for Ki67 (red) CD31 (green), and DAPI (blue). Scale bars represent 1 mm and 500 µm. E) Quantification of IHC analyses for cell nuclei density (i), % of pH3 (ii) and Ki67 (iii) positive cells, and CD31 intensity (iv) in HUVECs within regions 1 and 2 of 3D constructs (*n* = 4 / group). *p < 0.05, **p < 0.01, ***p < 0.001, and ****p < 0.0001.

Of note, the endothelial layers forming on the bioprinted channels showed mostly more than one layer of ECs (2‐3 layers), which is different from the monolayer of ECs lining the native human vasculature^[^ ^107^ ^]^ and could introduce a source of error in the bioprinted platform for faithful modeling of vascular tissue physiology. The increased thickness of endothelium could be attributed to several factors. Importantly, this could be due to the EC (over)growth, particularly in the bifurcation region (Figure 5H), which is in line with the PVS pathophysiology and supported by the IHC results on EC proliferation (**Figure** **6**). The uneven jagged surface of printed channels could also result in local entrapment of cells and formation of thicker EC clusters (cyan arrows, Figure 5H). In addition to the possibilities above, multi‐layer EC lining is commonly reported in bioengineered in vitro models of vascular tissues, typically due to the overseeding of constructs with cells to ensure full lumen coverage.^[^ ^108^, ^109^ ^]^ This challenge deserves more in‐depth research to further enhance the biomimicry and clinical relevance of bioprinted vascular models.

To examine the relevance of the established bifurcated PV model to the PVS pathophysiology, we conducted bulk RNA‐seq analysis on the cells collected from the proximal (region 1, target) versus distal (region 2, off‐target) regions within the perfused models following 10 days of culture (Figure S4, Supporting Information). Sequencing results of regions 1 and 2 in the in vitro models were compared against the patient‐derived PVS tissue sample. The ECs in the proximal region 1) demonstrated the closest gene expression profile to that of the human ex‐vivo PVS tissue (compared to healthy donor PV tissue obtained during transplant donation as baseline), while the cells in the distal region 2) showed noticeable differences. The more comparable trends in the expression of genes implicated in inflammation, cell‐ECM remodeling, and growth^[^ ^110^ ^]^ between bioprinted region (1) and the PVS patient tissue confirmed the greater chance of stenosis in the bifurcation region.

Once endothelial coverage and viability on the bioprinted PV channels were established, we next examined the therapeutic impact of drug‐loaded NPs on inhibiting HUVEC proliferation at the target site (region 1) in perfused 3D constructs (Figure 6). IHC analysis showed compact and relatively uniform endothelium covering both the top and bottom channel surfaces in all groups (Figure 6B,C). Of great significance, noticeable differences were observed in the morphology/assembly of ECs, lining on the top versus bottom surface of printed channels, where the top surface showed a greater number of ECs oriented perpendicular to the channel axis, branching into the GelMA matrix (white arrows in Figure 6B,C). This difference in EC alignment and increased branching may result in significant angiogenic behavior and can be attributed to several factors; the external static magnetic field and the force exerted by the magnet that was located at a short distance from the top lumen surface could result in pulling the ECs inward into the GelMA. The uptake of SPIONs by ECs could confer magnetic properties to the cells, and therefore, move the cells along the external magnetic flux direction (Figure 4, Figure S2, Supporting Information). This is consistent with recent works on efficient magnetic field‐guided delivery of magnetized cells (e.g., nanoparticle‐loaded human ECs^[^ ^111^ ^]^ and stem cells^[^ ^112^ ^]^). Increased HUVEC penetration into the GelMA and a more orthogonal cell orientation to the PV walls could be also partly regulated by the altered (higher) levels of WSS on the top channel surface, particularly at the bifurcation zone, as reported before^[^ ^42^, ^113^ ^]^ and predicted by CFD in this study (Figure 4). In addition, the distinctive bioprinted hydrogel ultrastructure between the top versus bottom parts of the PV channels could have contributed to the different cellular assembly and migration capacity. As revealed by SEM analysis of PV constructs, the DLP bioprinted channels consisted of an anisotropic porous, layered, and jagged structure, which, according to our (unpublished) observations, differs between the bottom, well‐supported half of constructs and top layers that sit on top of the hollow channel (including the possible gravity effect). The anisotropic structure/properties of DLP printed constructs, also reported by other groups,^[^ ^114^, ^115^ ^]^ could contribute to altered EC morphology in the top versus bottom lumen surfaces.

To probe the effect of targeted delivery of rapamycin on EC proliferation, we examined various proliferation makers, including Ki67 (Figure 6B,C) and pH3 (Figure 6D). Compared to off‐target, region 2 (outlet), targeted delivery of rapa‐SPIONs to the bifurcation (region 1) resulted in significantly (*P* < 0.05) reduced density of HUVECs (Figure 6E‐i). In comparison to the control 3D culture groups treated with no drug/SPIONs and treated with plain SPIONs, the targeted rapa‐SPION treatment also resulted in significantly (*P* < 0.05 and 0.01) reduced levels of pH3+ and Ki67+ cells in the bifurcation region (Figure 6E‐ii,iii). The average CD31 intensity (EC‐specific marker) decreased in the targeted region 1 (Figure 6E‐iv). These results confirmed the selective and effective role of magnetic field‐guided delivery of rapa‐SPION in inhibiting the endothelial growth, while causing minimal (insignificant) impact on cell behavior in the off‐target regions. Consistent with the data obtained in prior steps, no significant differences were observed in the EC density and proliferation between the no SPION and plain SPION groups.

## 4 Conclusion

The results from this study demonstrate a successful integration of several disciplines of science and technology, including nanotechnology and nanomedicine, functional biomaterials, high‐precision tissue biomanufacturing methods, and cell biology to develop a more effective and safer drug delivery approach with robust potential for clinical and translational applications. The combination of DLP‐based bioprinting and hybrid hydrogel‐based bioinks provides a promising platform for the establishment of high structural accuracy, tunability, throughput, and biomimicry in the patient‐specific in vitro models of vascular homeostasis and diseases. Compared to the conventional 2D culture models utilized here and elsewhere,^[^ ^116^, ^117^ ^]^ the developed 3D model integrated the biological, structural, mechanical, and physical features that more closely resemble those of the native vascular tissue. Using this engineered model, we demonstrated that a small external magnetic field could effectively target the drug‐loaded NPs onto the site of interest, minimizing the therapy's side effects in remote regions. While diminishing the need for extensive use of animal models, these in vitro platforms could play a pivotal role in studying and optimizing a large variety of factors that are involved in the magnetic field‐mediated delivery of therapeutics; these include the therapeutic selection (e.g., combination therapies), dosing, and duration, potential side effects, and the magnetic field strength, position, and duration. Such multidimensional in vitro studies could be of particular importance in teasing out the cellular and molecular mechanisms underlying the initiation, progress, and therapeutic interventions related to PV stenosis and restenosis.^[^ ^22^, ^23^, ^24^, ^25^ ^]^ While the use of SPIONs for targeted drug delivery has been established and reported by us^[^ ^39^, ^118^, ^119^ ^]^ and others,^[^ ^86^, ^89^ ^]^ this study introduces a new generation of 3D bioprinted, perfusable, in vitro platforms of human vasculature, with precise tuning and patient‐specificity capabilities, to test and optimize the delivery approaches in highly biomimetic 3D culture conditions. Future studies could further examine the more optimal dosage and regimen of SPION (or other nanomedicine) administration to ensure avoiding the excessive NP accumulation in the cells and toxicity.

Current study demonstrated the feasibility and efficacy of fabricating patient‐inspired pulmonary vasculature models, their uniform endothelialization, and dynamic culture within a custom‐designed chamber that enables applying highly controlled static magnetic fields. Future generations of these models could benefit from incorporating other key cellular components involved in the pulmonary vasculature homeostasis and stenosis, including smooth muscle cells, fibroblasts, and immune cells.^[^ ^3^, ^110^ ^]^ The efficacy of other types of nanocarrier/NP products, coupled with various therapeutic compounds, could be further tested in these platforms to examine and optimize such nanomedicines prior to moving to animal studies and/or clinical trials. More sophisticated perfusion systems could be also designed to allow for more physiologically relevant flow rates (3–4 L min^−1^ for the native human PVs^[^ ^92^ ^]^), while preserving the soft tissue material/cellular structures. Such enhancement would help to recapitulate generate the in vivo‐like flow hemodynamics within bioprinted channels and thus, enhance clinical relevance.^[^ ^94^, ^95^ ^]^ The bioprinted patient and injury‐specific models of PV/PVS can be fabricated in large numbers for high‐throughput experimental modeling of a variety of interventional procedures that are currently used in clinical settings, to enhance, optimize, or develop new techniques.^[^ ^36^ ^]^ Finally, the engineered vascular models could be adopted and applied more broadly to model a variety of processes involved in human vascular homeostasis and diseases, such as atherosclerosis, arterial hypertension, and valve disease.

## Conflict of Interest

The authors declare no conflict of interest.

## Supporting information

Supporting Information

Supplemental Video 1

Supplemental Video 2

Supplemental Video 3

Supplemental Video 4

Supplemental Video 5

Supplemental Video 6

Supplemental Video 7

Supplemental Video 8

Supplemental Video 9

Supplemental Video 10

Supplemental Video 11

Supplemental Video 12

## Data Availability

The data that support the findings of this study are available from the corresponding author upon reasonable request.
