# Supplementary material for: Targeted Rapamycin Delivery via Magnetic Nanoparticles to Address Stenosis in a 3D Bioprinted in Vitro Model of Pulmonary Veins
Source: Adv Sci (Weinh). 2024 May 2;11(26):2400476. doi: 10.1002/advs.202400476 (PMC11234432; doi:10.1002/advs.202400476)
Supplement: Supplementary file 1 — Supporting Information [file ADVS-11-2400476-s008.pdf]

ADVANCED SCIENCE
Open Access

Supporting Information

for *Adv. Sci.*, DOI 10.1002/advs.202400476

Targeted Rapamycin Delivery via Magnetic Nanoparticles to Address Stenosis in a 3D Bioprinted in Vitro Model of Pulmonary Veins

Liqun Ning, Stefano Zanella, Martin L. Tomov, Mehdi Salar Amoli, Linqi Jin, Boeun Hwang, Maher Saadeh, Huang Chen, Sunder Neelakantan, Lakshmi Prasad Dasi, Reza Avazmohammadi, Morteza Mahmoudi, Holly D. Bauser-Heaton and Vahid Serpooshan**

Supplementary Information

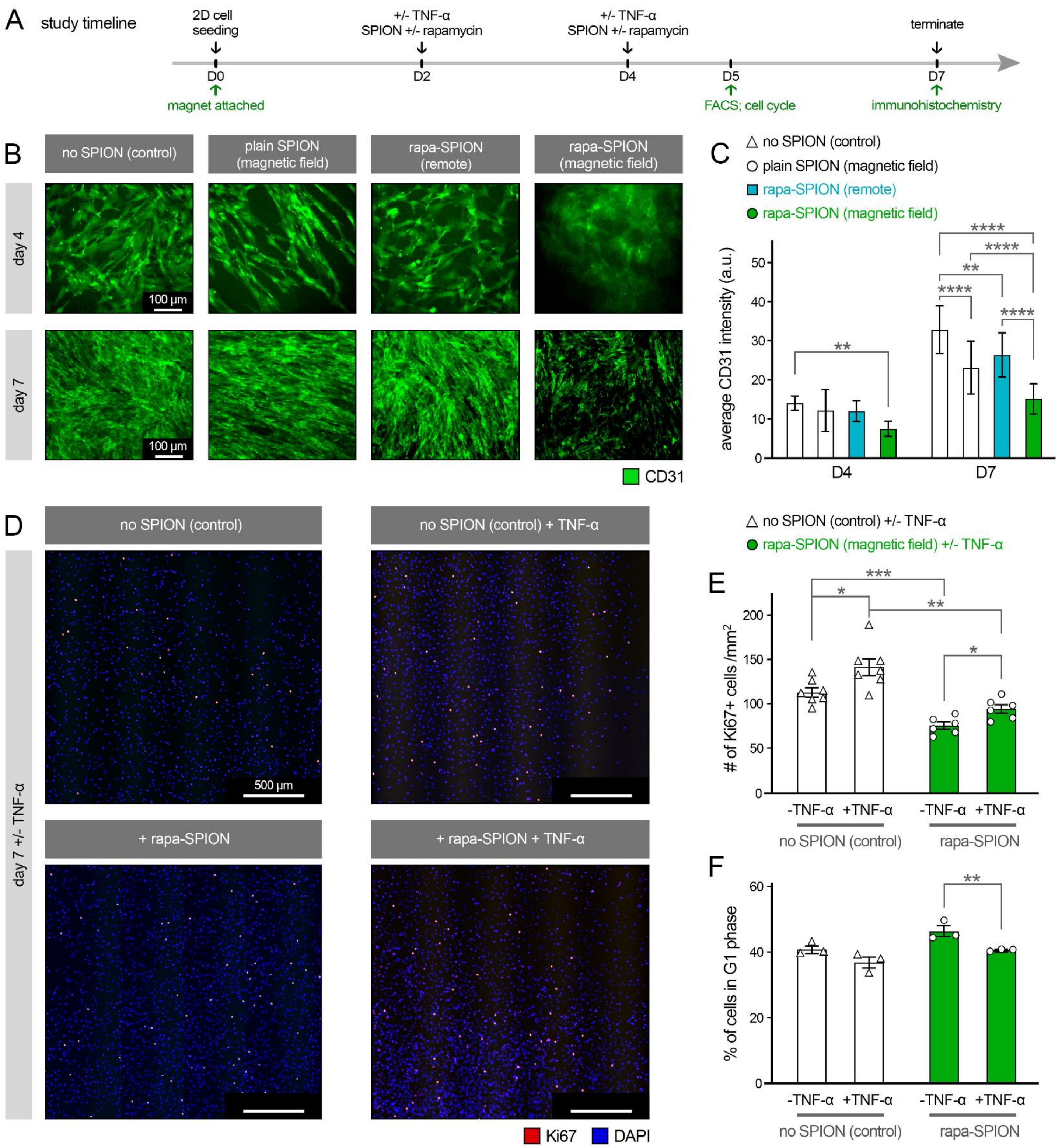

Figure S1. Evaluating the effect of magnetic targeting of rapamycin loaded superparamagnetic iron oxide nanoparticles (rapa-SPIONs) on the monolayer culture of human umbilical vein endothelial cells (HUVECs) in the absence and presence of TNF-α. **A**: Study timeline. **B-C**: Targeted delivery of rapa-SPIONs to the 2D HUVEC cultures resulted in a significant decrease in the expression level of EC-specific CD31 (green) in the magnetic field at days 4 and 7 of culture, when compared to the control (no SPION), plain SPION cultures, and cells in the remote (off-target) areas (n = 3 per group). Scale bars represent 100 µm. **D-F:** The effect of targeted rapa-SPION treatment on HUVEC proliferation was also examined in the absence and presence of TNF-α. **D:** Immunostaining of HUVECs at day 7 of culture obtained for the four experimental groups. DAPI staining (blue) labeled the nuclei and Ki67 staining (red) was used to label proliferating cells. Scale bar in all images represent 500 µm. **E:** Quantification of Ki67+ cells, normalized by the surface area, for all experimental groups. A minimum sample size of n = 6 was used for each group. **F:** Percentage of HUVECs in the G1 phase, obtained via cell cycle analysis for the HUVEC cultures in the absence and presence of TNF-α (n = 3 per group). *: $p < 0.05$, **: $p < 0.01$, ***: $p < 0.001$, and ****: $p < 0.0001$.

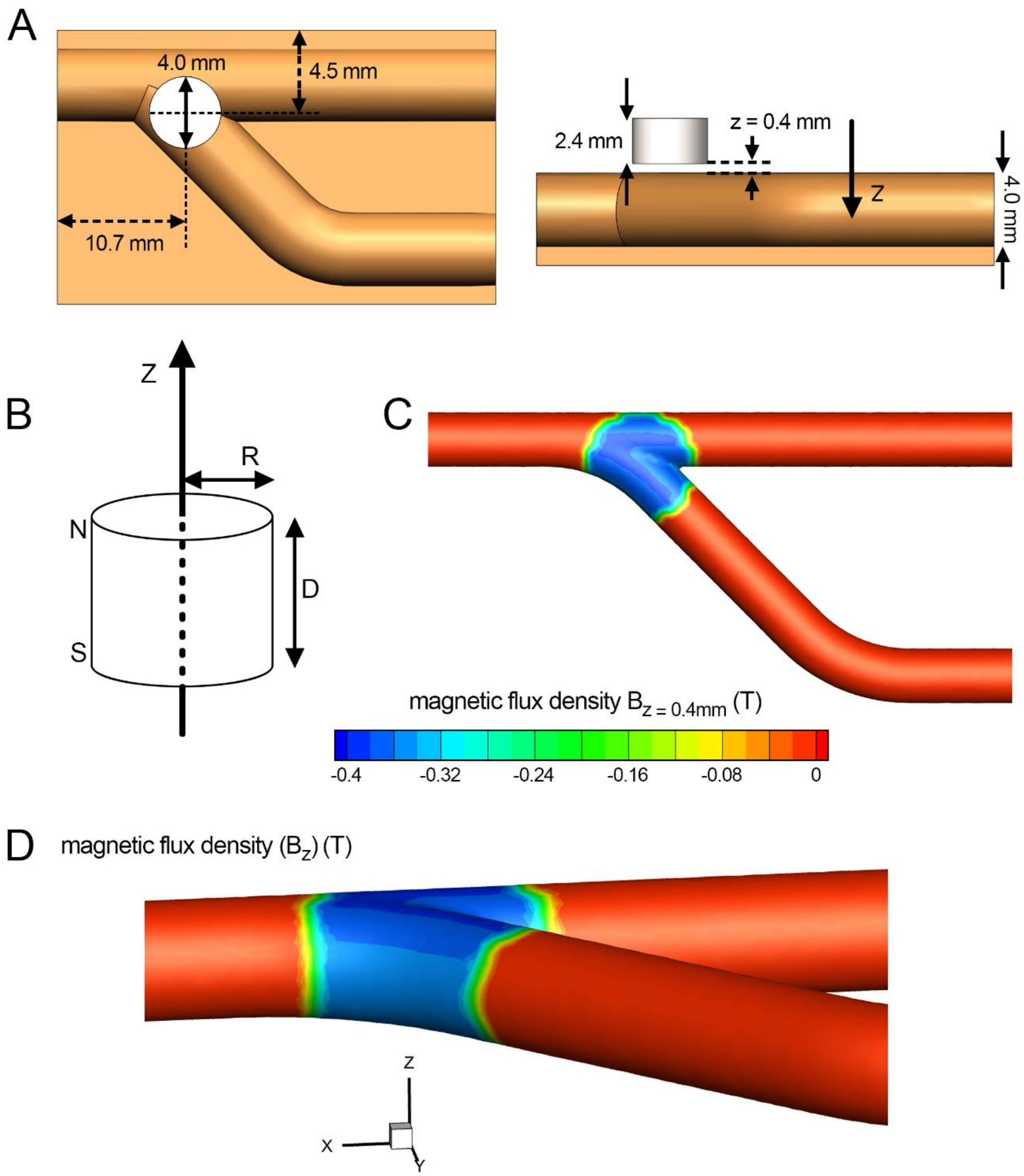

Figure S2. Geometric configuration of the magnet withing perfusion bioreactor assembly to target nanoparticles (NPs). A: Schematic diagrams illustrating the magnet location and important dimensional parameters used in the study. **B:** Magnetic flux density (B_z = 0.4 T) was calculated based on the geometric parameters illustrated, along with the inherent parameters extracted for the magnet, using Equation 5 (see methods). **C:** B_z distribution in the x-y plane at the top lumen surface of channels ($z = 0.4\ mm$), confirming the 0.4 T magnetic flux at the center of the magnet, which was accurately focused onto the vascular bifurcation zone. **D:** B_z in the z direction (towards the magnet), demonstrating a rather uniform magnetic field flux across the lumen.

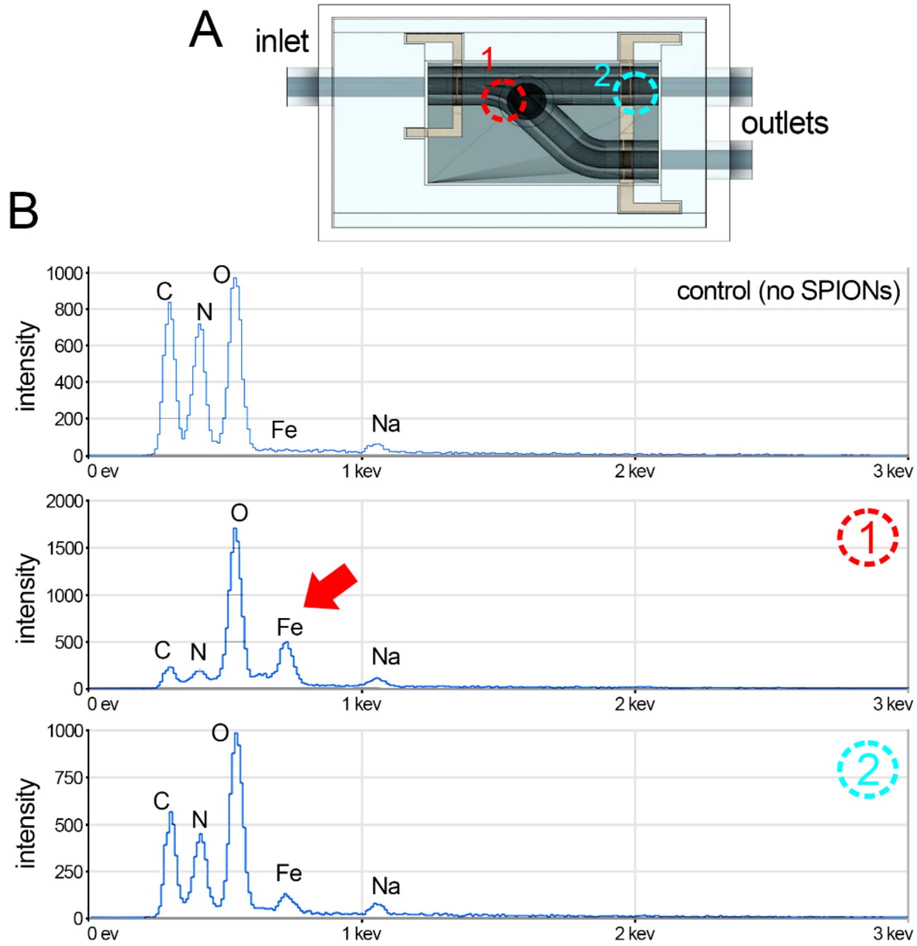

Figure S3. X-ray energy dispersive spectroscopy (EDS) and scanning electron microscopy of bioprinted vascular constructs to assess nanoparticle (NP) targeting. A: Schematic diagrams illustrating the vascular tissue geometry and the regions 1 (magnetic targeting region) and 2 (off-target region) that were examined via EDS. **B:** EDS spectra of the control sample (top row), cultured without superparamagnetic iron oxide NPs (SPIONs), as well as regions 1 and 2 from constructs (middle and bottom rows) perfused with SPIONs. The Fe peak came from the SPIONs circulated through the printed channels.

A

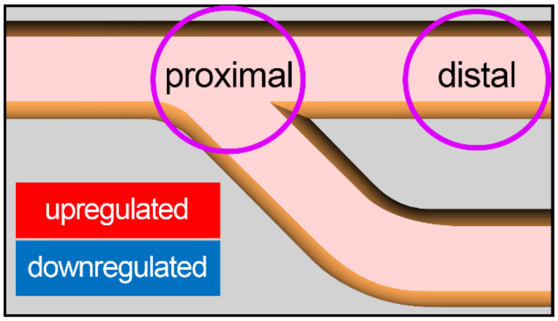

B

Gene	distal	proximal	PVS tissue
TGFBR1	-0.77	0.03	1.03
TNFa	-0.56	-0.76	1.00
IL6	-0.49	-0.25	0.44
TLR5	-0.22	0.03	0.39
VIM	-0.66	-0.54	0.34
COL3A1	0.09	0.61	0.32
NOS3/eNOS	-0.64	0.75	0.29
FN1	-1.54	-0.28	0.26
MAPK3/ERK1	-1.36	0.86	0.25
BMP4	-0.09	0.41	0.24
NFKB1	-0.68	-0.28	0.18
TGFB1	-1.11	0.11	0.15
NFKB2	-1.05	-0.14	0.14
BMPR2	-0.56	0.17	0.12
VEGFR1	-1.15	0.10	0.07
MAPK1/ERK2	-0.83	-0.36	-0.09
FGFR	-0.71	-0.25	-0.10
SMAD2	-0.74	0.11	-0.15
BMP7	-1.16	0.90	-0.32
TGFB2	-0.49	0.53	-0.51
GATA4	-0.76	0.98	-0.69

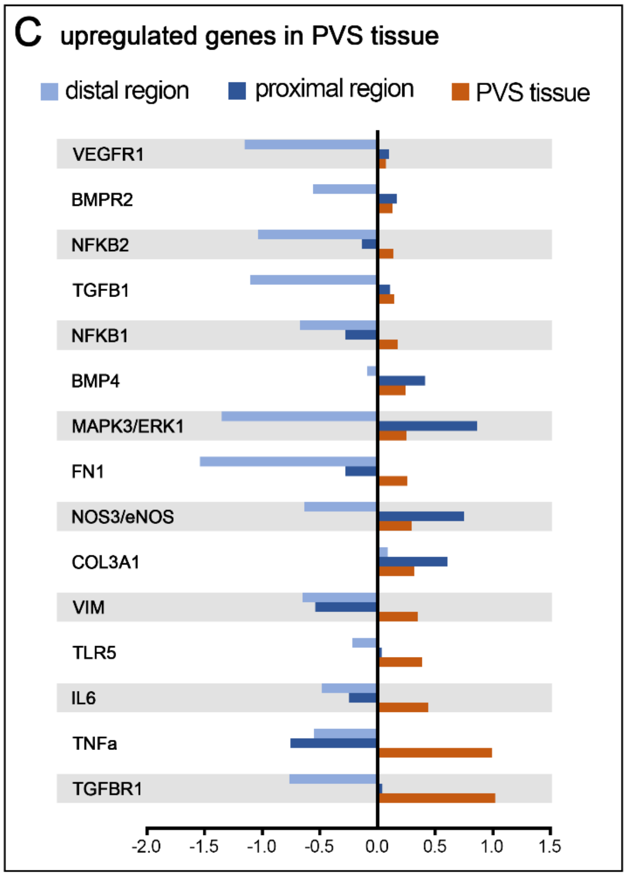

Figure S4. Bulk RNA sequencing analysis of cells collected from various regions within 3D bioprinted models of pulmonary veins (PVs) versus patient derived tissue. A: Schematic diagram illustrating the distinct vascular regions used to collect cells and extract RNA from, including the proximal (target) region and the distal (off-target) region. **B-C:** A selection of upregulated (red) and downregulated (blue) genes obtained in comparison of cells collected from proximal and distal regions of bioprinted models, together with tissue obtained ex-vivo from a patient with PV stenosis (PVS). Panel (**C**) shows the fold-change in the expression of several upregulated genes with important roles in the PVS pathophysiology as compared to healthy PV from donor control.
